# Muscular responses to upper body mediolateral angular momentum perturbations during overground walking

**DOI:** 10.3389/fbioe.2025.1509090

**Published:** 2025-04-11

**Authors:** Omid Mohseni, Andrew Berry, Christian Schumacher, Andre Seyfarth, Heike Vallery, Maziar A. Sharbafi

**Affiliations:** ^1^ Lauflabor Locomotion Laboratory, Institute of Sport Science, Centre for Cognitive Science, Technical University of Darmstadt, Darmstadt, Germany; ^2^ Measurement and Sensor Technology Group, Department of Electrical Engineering and Information Technology, Technical University of Darmstadt, Darmstadt, Germany; ^3^ Delft Biorobotics Lab, Faculty of Mechanical, Maritime and Materials Engineering, Delft University of Technology, Delft, Netherlands; ^4^ Faculty of Mechanical Engineering, Rhine-Westphalia Technical University of Aachen, Aachen, Germany

**Keywords:** balance control, perturbed walking, perturbation-invoked muscle activity, postural adjustments, control moment gyroscope

## Abstract

Adaptive motor control and seamless coordination of muscle actions in response to external perturbations are crucial to maintaining balance during bipedal locomotion. There is an ongoing debate about the specific roles of individual muscles and underlying neural control circuitry that humans employ to maintain balance in different perturbation scenarios. To advance our understanding of human motor control in perturbation recovery, we conducted a study using a portable Angular Momentum Perturbator (AMP). Unlike other push/pull perturbation systems, the AMP can generate perturbation torques on the upper body while minimizing the perturbing forces at the center of mass. In this study, ten participants experienced trunk perturbations during either the mid-stance or touchdown phase in two frontal plane directions (ipsilateral and contralateral). We recorded and analyzed the electromyography (EMG) activity of eight lower-limb muscles from both legs to examine muscular responses in different phases and directions. Based on our findings, individuals primarily employ long-latency hip strategies to effectively counteract perturbation torques, with the occasional use of ankle strategies. Furthermore, it was found that proximal muscles, particularly the biarticular Rectus Femoris, consistently exhibited higher activation levels than other muscles. Additionally, in instances where a statistically significant difference was noted, we observed that the fastest reactions generally stem from muscles in close proximity to the perturbation site. However, the temporal sequence of muscles’ activation depends on the timing and direction of the perturbation. These findings enhance reflex response modeling, aiding the development of simulation tools for accurately predicting exogenous disturbances. Additionally, they hold the potential to shape the development of assistive devices, with implications for clinical interventions, particularly for the elderly.

## 1 Introduction

Maintaining stable balance during walking is a challenging motor task that involves a highly coordinated interplay between the central nervous system, muscles, and sensory feedback. This fundamental aspect of human locomotion is a remarkable feature of the human neuromuscular system and is essential for preventing falls and ensuring safe and efficient movement (Reimann et al., 2018b). Understanding the underlying mechanisms of coordinated muscle actions in response to perturbations is of great importance for elucidating the complex nature of balance control and for developing strategies to prevent balance-related injuries.

When confronted with perturbations, such as unexpected external forces or disturbances to the body’s center of mass (CoM), the neuromuscular system must rapidly adapt to maintain balance. Coordinated muscle actions play a critical role in this process by selective recruitment and modulation of muscle activity across different joints and limbs (Horak and Nashner, 1986; Moore et al., 1988). The activation patterns of muscles are carefully orchestrated to generate appropriate joint torques and control the body’s orientation. These muscle activations occur in a precise temporal sequence and are influenced by miscellaneous factors including, but not limited to, perturbation type (moment (van Mierlo et al., 2022), forces or pushes (Hof A. and Duysens J., 2018; Fang and Lerner, 2023; Zhu et al., 2023), slip (Tang et al., 1998; O’Connell et al., 2016), stumbling over obstacles (Schillings et al., 2000), falls (Cano Porras et al., 2021)), perturbation specification (intensity (O’Connell et al., 2016; Hof A. and Duysens J., 2018), timing (Golyski et al., 2022; Hof A. and Duysens J., 2018; Schillings et al., 2000), direction (Hof A. and Duysens J., 2018), duration, location, plane), individual characteristics (mass, height, gender, age (Boisgontier et al., 2013; Rosenblum et al., 2022; Afschrift et al., 2019), impairment (Garland et al., 2009; Willaert et al., 2023), prior experience (Marigold and Patla, 2002), muscle tone (Kaminishi et al., 2021)), and specific task requirements (sitting (Zedka et al., 1998), standing (Schumacher et al., 2019), walking (Vlutters et al., 2018), running (Van Woensel and Cavanagh, 1992; Snyder et al., 2012), dual task (Rosenblum et al., 2022)).

Emerging research has shed some light on the complex muscle coordination strategies to counteract perturbations and maintain balance (Schumacher et al., 2019; Firouzi et al., 2024). However, much previous research has focused on muscle coordination in standing balance with force-based mechanical perturbation types that affected the CoM excursions. It is, therefore, crucial to extend the scope of these investigations beyond standing (Taleshi et al., 2022; Borkowski and Błażkiewicz, 2023) and consider the specific challenges associated with walking (Reimann et al., 2018a). Further, maintaining balance during gait in the face of mediolateral (ML) perturbations is known to be more challenging as compared to anterior-posterior ones (Williams et al., 1997; Bauby and Kuo, 2000; Borkowski and Błażkiewicz, 2023; Alili et al., 2023) and requires active control (Brough et al., 2021; Firouzi et al., 2022). When confronted with frontal-plane perturbations, more pronounced effects (e.g., higher rate of center of pressure changes (Henry et al., 1998) or increased ground reaction force (Tokur et al., 2020)) are exhibited, which may lead to larger postural misalignments than sagittal perturbations (Henry et al., 1996; Tokur et al., 2020). Foot placement, for instance, as an extensively studied balance strategy (Bruijn and Van Dieën, 2018; Vlutters et al., 2016; Hof et al., 2007; 2010a; Hof and Duysens, 2013), plays a significant role in countering perturbations and maintaining balance in the frontal plane compared to the sagittal plane (Van Mierlo et al., 2021). Given the significance of balance in the frontal plane during walking, further research is imperative to investigate the associated muscle response strategies, particularly with novel perturbation scenarios that have not been explored before.

Prior investigations focusing on unexpected perturbed walking in the ML direction have yielded valuable insights into the involvement of muscles in coping with perturbations and recovery strategies. For instance, ML platform translations necessitate heightened reactive tibialis anterior activity as an ankle strategy (Afschrift et al., 2019). Failure to implement this ankle strategy results in a shift to a stepping strategy (Afschrift et al., 2019). In response to perturbations in medial and lateral foot placement, both hip and ankle strategies are employed to regulate the overall angular momentum of the body (Brough et al., 2021). By intermittently perturbing the swing leg either medially or laterally, it was found that humans modulate swing-phase gluteus medius muscle activity in response to the mechanical state of the contralateral leg to control ML foot placement (Rankin et al., 2014). Lateral perturbations like pushes or pulls during gait are dealt with through a combination of a stepping strategy and a lateral ankle strategy (Hof et al., 2010a). Such perturbations elicit automatic muscle responses in the abductor muscle gluteus medius, resulting in an outward foot placement strategy in the subsequent step, with distinct response patterns and phase dependencies (Hof and Duysens, 2013). In an attempt to explore the connection between muscle activities and balance mechanisms, it was discovered that the contributions of muscles surrounding the ankle, in response to push and pull forces, elicit a braking reaction and generate an ML ankle strategy (Hof A. and Duysens J., 2018). A common thread among many of these studies is the predominant use of lateral perturbations induced by platform translation (Afschrift et al., 2019) or pulling/pushing at either the pelvis or shoulder (Borkowski and Błażkiewicz, 2023). Perturbations of this nature pose a challenge to the translational motion of the center of mass and indirectly disrupt the body’s angular momentum, necessitating intricate corrective actions that may engage multiple response mechanisms.

**FIGURE 1:**
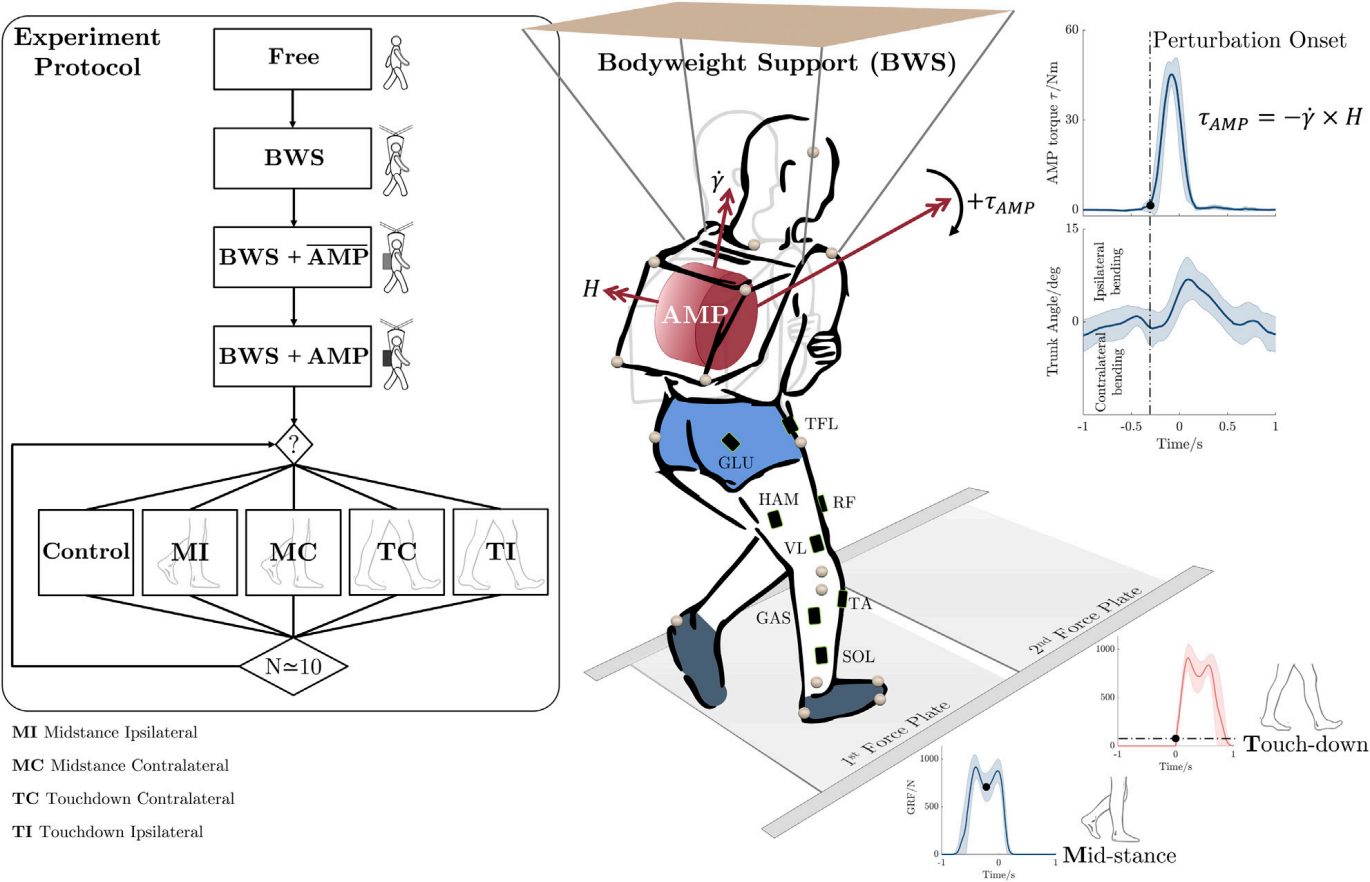
Perturbation protocol and the experimental setup. The participants participated in four walking conditions: *Free*, which represents normal overground walking; *BWS*, where a suspension system is used; $BWS+\bar{AMP}$ , involving a suspension system with a deactivated angular momentum perturbator (AMP); and lastly *BWS + AMP* with a bodyweight suspension system with an activated AMP. In the latter condition, the participants experience four types of frontal perturbations: *MI* and *MC* occurring on the first force plate, and *TC* and *TI* occurring on the second force plate. An exemplary perturbation scenario (*MI*) is illustrated on the right, showing the orientation of the angular momentum vector (H) , gimbal rotation (γ) , output gyroscopic moment $\left(\tau_{\mathrm{AMP}}\right)$ and its effect on the upper body’s roll bending. The locations of motion capture markers on the body and AMP, and EMG sensors for one leg are indicated. **GLU** Gluteus Maximus, **TFL** Tensor fasciae latae, **HAM** Biceps femoris long head, **RF** Rectus femoris, **VL** Vastus lateralis, **GAS** Gastrocnemius lateralis, **SOL** Soleus, **TA** Tibialis anterior.

In this study, we utilize an angular momentum perturbator (AMP) equipped with a control moment gyroscope (CMG) as the perturbation device. The AMP facilitates the manipulation of angular momentum by swiftly altering the orientation of a spinning wheel, thereby applying perturbation torques. Such a perturbation device induces minimal effects on CoM excursions (Lemus et al., 2017) (Figure 1). One of the primary advantages of AMP over other perturbation devices lies in its capacity to specifically target angular momentum regulation during walking, thereby distinguishing between linear and angular momentum modulation—a distinction often overlooked in prior studies. This study leverages the AMP to isolate and investigate the effects of pure rotational perturbations on balance control, providing a unique opportunity to understand the specific motor strategies employed in response to angular momentum disturbances. While such pure rotational perturbations may not directly correspond to common daily activities, they allow for a controlled examination of balance recovery mechanisms that are otherwise difficult to isolate in more complex, real-world scenarios. Moreover, rotational torques can be a predominant factor in certain perturbations encountered in everyday life, such as maintaining balance on a moving vehicle or when carrying uneven loads, where the rotational component significantly challenges stability. By focusing on these isolated perturbations, this research fills a critical gap in our understanding of human motor control during gait. This approach is particularly relevant for populations with impaired motor coordination, such as the elderly and post-stroke patients (Honda et al., 2019; Nott et al., 2014) who may disproportionately rely on distinct angular momentum compensation strategies. Research indicates that recovering balance from angular momentum perturbations may take precedence over restoring balance from linear momentum disturbances (Horak et al., 1997; van Mierlo et al., 2022). Failure to regulate whole-body angular momentum increases the risk of falls, emphasizing the need to understand how healthy individuals recover from whole-body angular momentum perturbations. Through analyzing human responses to angular moment perturbations produced by AMP, we can better understand balance-related motion inefficiency and enhance the design and development of rehabilitation techniques and assistive technology. Additionally, AMP offers advantages such as the assessment of overground walking, surpassing motorized treadmill setups by preserving optical flow. Its portability eliminates mechanical linkages between the body and the physical environment, reducing confounding factors related to mechanical constraints on the body. Moreover, AMP allows unrestricted movement of biological joints. These features collectively position AMP as an excellent candidate for both perturbation and balance-assistive purposes (Sterke et al., 2023).

In our experimental setup, the AMP is incorporated as a backpack, introducing perturbing torques to the upper body of unimpaired adults during overground gait. Perturbations are randomly triggered at two different instances of the gait cycle (i.e., mid-stance and touchdown) and in both medial and lateral directions (see Figure 1). Notably, our use of a CMG as a perturbation source differentiates the present work from previous studies that have investigated force-based mechanical perturbations during human walking. Here, we aim to enhance our understanding of the multifaceted nature of human balance recovery during overground walking by investigating activation patterns of major lower-limb muscles. We examine the relative involvement of biarticular and monoarticular leg muscles along with the adopted reactive strategies in response to these upper-body gyroscopic moment perturbations. We hypothesize that muscle reactions may be influenced by perturbation proximity, indicating that muscles closest to the perturbation source will exhibit more rapid and pronounced reactions (Hypothesis 1). Consequently, we expect the utilization of hip strategies and corresponding responses in the hip muscles. Based on previous findings suggesting the importance of biarticular leg muscles in locomotion (Schumacher et al., 2020) and in standing balance control (Schumacher et al., 2019), here we hypothesize that thigh biarticular muscles exhibit critical involvement in generating reactive balance adjustments (Hypothesis 2). We further hypothesize that the muscular response sequence follows a proximal-to-distal pattern originating from the site of the perturbation (Hypothesis 3). This hypothesis explores the manner in which supplementary muscles are enlisted along segmental chains. In situations where the proximal joint alone proves inadequate in offsetting the perturbation’s effects, adjacent muscles are sequentially recruited. This inquiry delves into the fundamental strategies governing the recruitment of muscles in the coordination of multi-segment movements.

## 2 Methods

### 2.1 Angular momentum perturbator (AMP)

The AMP , worn as a backpack, incorporates a CMG that manipulates the angular momentum of an internal flywheel to exert torques. The flywheel is mounted on a motorized gimbal frame, enabling reorientation with respect to the wearer. The AMP used in this study was slightly modified from its previous version (Schumacher et al., 2019); it weighs 16 kg and has an estimated peak gyroscopic moment of 49 N m (Supplementary Figure S4). All sensors and motors interface with an on-board microcontroller (STM32-H405, Olimex, Bulgaria) and relay information via a wired RS485 connection to an off-board PC executing the high-level control loops at a fixed sampling rate of 500 Hz (implemented in Simulink Real-Time R2016b, The Mathworks, Natick, United States).

The AMP applies torques to the wearer $\left(\tau_{AMP}\right)$ by manipulating the angular momentum (H) of the flywheel. By rotating the gimbal or the wearer’s trunk, gyroscopic torques proportional to the gimbal’s angular velocities $\left(\dot{\gamma }\right)$ and the wearer’s trunk angular velocity $\left(\omega =\left[\omega_{x},\omega_{y},\omega_{z}\right]\right)$ are generated. The gimbal motor adjusts $\dot{\gamma }$ by applying a torque $\left(\tau_{g}\right)$ , but this alters the magnitude of H , resulting in an opposite reaction torque experienced by the wearer. The total torque comprises these components combined; see Equation 1. By rotating the gimbal to generate controlled gyroscopic torques, the direction of $\left(\tau_{AMP}\right)$ changes in conjunction with the gimbal angle (γ) . In the human-fixed frame (with unit vectors $\left({\hat{e}}_{x},{\hat{e}}_{y},{\hat{e}}_{z}\right)$ attached to the frame (x,y,z) ), the total perturbation torque $\tau_{AMP}$ encompasses components in all three directions as in Equation 2.

$$\tau_{AMP}\left(t\right)=-\dot{H}\left(t\right)=-\underset{\text{gyroscopic effect}}{\underbrace{\left(\dot{\gamma }\left(t\right)+\omega \left(t\right)\right)\times H\left(t\right)}}-\underset{\text{gimbal motor}}{\underbrace{\tau_{g}\left(t\right)}}$$ (1)

$$=-\underset{\text{pitch}}{\underbrace{\tau_{t}\left(t\right)\;\cos\left(\gamma \left(t\right)\right){\hat{e}}_{x}}}-\underset{\text{roll}}{\underbrace{\tau_{t}\left(t\right)\;\sin\left(\gamma \left(t\right)\right){\hat{e}}_{y}}}+\underset{\text{yaw}}{\underbrace{\left(\omega_{t}\left(t\right)H\left(t\right)-\tau_{g}\left(t\right)\right){\hat{e}}_{z}}}$$ (2)

$$\approx -\dot{\gamma }\left(t\right)H\cos\left(\gamma \left(t\right)\right){\hat{e}}_{x}-\dot{\gamma }\left(t\right)H\sin\left(\gamma \left(t\right)\right){\hat{e}}_{y}+\left(\omega_{x}\left(t\right)H\cos\left(\gamma \left(t\right)\right)-\tau_{g}\left(t\right)\right){\hat{e}}_{z},$$

where $\tau_{t}\left(t\right)=\left(\dot{\gamma }\left(t\right)+\omega_{z}\left(t\right)\right)H\left(t\right)\approx \dot{\gamma }\left(t\right)H\left(t\right)$ and $\omega_{t}\left(t\right)=\omega_{x}\left(t\right)\cos\left(\gamma \left(t\right)\right)+\omega_{y}\left(t\right)\sin\left(\gamma \left(t\right)\right)\approx \omega_{x}\left(t\right)\cos\left(\gamma \left(t\right)\right)$ non-bold variables represent the scalar magnitudes of vector quantities mentioned earlier. The magnitude of the flywheel’s angular momentum remained nearly constant during all experiments (H(t)≈H) .

For the desired perturbations, the perturbation torque $\tau_{AMP}$ followed a symmetric trapezoidal profile. It had a peak torque of 60 Nm and rise, hold, and fall times of 100 ms each. To generate these perturbations, a gimbal motor torque $\left(\tau_{g}\right)$ of approximately 12 Nm was required.

### 2.2 Experimental protocol

Four consecutive sets of measurements were conducted as part of the experimental protocol. In the first setting, the participants were asked to walk at their preferred speed on the gait track (width 500 mm, length 2×600+2×1806= 4,812 mm) without wearing the AMP or a safety harness for 10 trials (‘Free’). In the second setting, they repeated the same task but wore a body-weight support safety harness for 10 trials (‘BWS’). In the third setting, they repeated the same task for another 10 trials while wearing the AMP with a spinning flywheel, but no active torque perturbations were given (‘BWS+ $\bar{AMP}$ ’). These three initial conditions—‘Free’, ‘BWS’, and ‘BWS+ $\bar{AMP}$ ’—were designed to evaluate whether the addition of the AMP or the use of the body-weight support system would influence kinematics or ground reaction force (GRF) characteristics before proceeding with the perturbation studies. Since the primary focus of this study is on EMG analysis, an in-depth discussion of these conditions is not provided in the main text but is included in the Supplementary Materials. In the final setting, a series of 48 trials were executed involving 40 randomized trials of active torque perturbation of the AMP (‘BWS + AMP’) and eight trials that lacked a perturbation (‘Control Trials’). The order of these 48 trials was randomized per participant using a computer randomization algorithm to prevent anticipation of perturbations and ensure unbiased comparisons. The 40 perturbation trials consisted of four conditions with 10 repetitions each with one gimbal position and one intensity level (unchanged for all participants): Contralateral and ipsilateral torque directions in the frontal plane at either midstance of the right leg or touchdown of the left leg. The AMP applied a perturbation on either the one or two force plate, corresponding to the three or four steps, respectively. Overground walking continued for another one or two steps before slowing to a stop (Supplementary Figure S1). The perturbations were not scaled with respect to each participant’s anthropometric features (e.g., mass, height). This was done to 1) maximize the degree of postural threat, 2) retain similar operating characteristics of the AMP, and 3) avoid incorrect assumptions about scaling with respect to anthropometric features.

In order to avoid fall-related injuries during the perturbations, a safety harness was consistently employed whenever the Gyro was worn. This harness was connected to the RYSEN body weight support system (Motekforce Link, Amsterdam, Netherlands), which possesses the ability to actively detect and halt the participant’s falling movements (Plooij et al., 2018). To avoid vertical unloading forces during the measurements, the RYSEN system was set at its lowest assistance level. (see Supplementary Figure S16 for a comparison between ‘Free’, ‘BWS’, and ‘BWS+ $\bar{AMP}$ ’).

### 2.3 Data collection and processing

#### 2.3.1 Participants

The study involved sixteen adult participants, with one female and fifteen males. The inclusion of only one female participant in our study was primarily due to participant availability. All participants willingly volunteered to take part in the research during the summer of 2018. Prior to the experiment, they provided written informed consent. The experimental protocol was approved by the Human Research Ethics Committee of the Delft University of Technology (Project ID: 350), and all procedures were conducted in accordance with the relevant guidelines and regulations of the Declaration of Helsinki. Before the experiment commenced, participants gave their consent and completed the revised Waterloo Footedness Questionnaire (Elias et al., 1998), which aimed to evaluate limb dominance in relation to stabilization tasks.

We collected EMG, kinetic, and kinematic data from the participants (Mohseni et al., 2024a). All measurement devices, including the data logging of the AMP, were synchronized by a manual trigger signal. Prior to data processing, two participants were excluded from the data analysis due to varying stepping patterns. A further four participants were excluded due to missing EMG data or missing marker data which hindered the calculation of gait events or inverse kinematics. Only the data of the remaining 10 participants (one female), of age 34.1±14.2 years (Mean±SD) , weight 76.1±12.4 kg, and height 1.79±0.08 m, were considered for further analysis. All participants have the same limb preference and step with the same leg onto the first force plate.

#### 2.3.2 Measured data and preprocessing

Sixteen surface EMG electrodes (Trigno, Delsys Inc., Natick, United States) were used to record at 2000 Hz the electrical activity of relevant leg muscles. The set of electrodes was placed on the following muscles of each leg: tibialis anterior (TA), soleus (SOL), gastrocnemius lateralis (GAS), vastus lateralis (VL), rectus femoris (RF), biceps femoris long head (HAM), tensor fasciae latae (TFL) and gluteus maximus (GLU) (Supplementary Figure S2). Out of the eight lower-limb muscles recorded, three muscles are directly involved in movements in the frontal plane: GLU (responsible for hip abduction and lateral rotation), TFL (involved in hip abduction and medial rotation), and TA (associated with foot inversion). It’s important to note that frontal plane rotation can be influenced not only by muscles that directly control joints with rotational freedom in that plane but also indirectly through the horizontal offset of the supporting limbs. To ensure good electrical connectivity, the skin preparation adhered to the guidelines recommended by SENIAM (Hermens, 1999). Once the electrodes were attached, the locations were examined for voluntary muscle signals and minimal noise levels. The raw electromyography (EMG) signals underwent several filtering steps. Initially, a band-pass filter was applied with cut-off frequencies of 40 Hz (high-pass) and 450 Hz (low-pass). Subsequently, an IIR notch filter was implemented at 50 Hz to eliminate any interference. Lastly, a zero-lag fourth-order Butterworth low-pass filter was employed for further signal refinement. For each muscle and participant, the filtered EMG signals were normalized by the average peak of EMG in ‘Control Trials’.

Two force plates with built-in amplifiers (9260AA6, Kistler Holding AG, Winterthur, CH) located in the middle of the walkway (corresponding to the three or four steps) with data acquisition units (5695B, Kistler Holding AG, Winterthur, CH) were used to measure individual GRF of each leg at a frequency of 1,000 Hz. The GRF data was anti-aliasing low-pass filtered in real-time with a third-order analog Butterworth filter with a cut-off frequency of 500 Hz. The onset of perturbations was triggered via the force plates. Contact with either force plate was detected when the vertical force exceeded 2% of the participant’s body weight. Mid-stance was detected when the vertical force increased for 10 ms between 300 ms and 600 ms after initial contact with the force plate. Touchdown was detected as the first instance of contact with the second force plate.

To collect kinematic data, an inertial measurement unit (IMU) measuring at 500 Hz (MPU-9250, InvenSense, San Jose, United States) was integrated into the AMP. Additionally, a motion capture system from Qualisys (Gothenburg, Sweden) was utilized. Kinematic data were recorded at a frequency of 200 Hz. Nineteen reflective markers were positioned on specific anatomical landmarks, including the tragion (TRA), seventh cervical vertebrae (C7), acromion (ACR), greater trochanter (GTR), lateral femoral condyle (LFC), fibulare (FIB), lateral malleolus (LM), calcaneus (CAL), first metatarsal head (MT1), and fifth metatarsal head (MT5). Four extra markers were placed on the stationary and rigid frame of the AMP in a configuration that approximated the mean position of the AMP’s CoM (Supplementary Figure S2). The QTM software was utilized for marker labeling and interpolating missing samples in the marker data through the polynomial gap-filling tool. To automatically determine the timing of touchdown and toe-off, foot velocity algorithm (O’Connor et al., 2007) was employed.

Data processing was conducted using Matlab R2020b (MathWorks Inc., Natick, United States). OpenSim 4.3 was employed to scale a 23-DoF full-body model, incorporating 92 musculotendon actuators representing 76 muscles for each participant (Delp et al., 2007). Additionally, the gyroscopic backpack was incorporated into the model using the four AMP markers and the calculated AMP inertia. The OpenSim inverse kinematics, inverse dynamics, and analysis tool were employed to extract joint moments as well as the positions, velocities, and orientations of the CoM for both the whole body and individual segments. While these analyses were performed to support the study, the primary focus of this paper is on EMG analysis; thus, detailed results of the kinematics and GRF analyses are presented in the Supplementary Materials (Supplementary Figures S3, 6–16), with only key findings briefly summarized in the Results section.

#### 2.3.3 Computing outcome measures

The mean absolute value (MAV) of EMG as a representation of the level of muscle activity was calculated by averaging over the absolute EMG data for all four different perturbation cases. This value was then subtracted from the MAV of control trials and expressed as a percentage.

The excitatory response of a muscle to the gyroscopic perturbation was identified by observing a significant increase in muscle activity surpassing one standard deviation above the grand average of control trials. This heightened activity persisted for a minimum of 30 ms (Tang et al., 1998). The duration of the elevated muscle activity was defined as the temporal span encompassing the onset and offset of the increased activity. The endpoint of the burst was determined when the perturbed trial’s muscle activity dropped below the average of the control trials for at least 30 ms.

For the statistical analysis, linear mixed-effects models (LMMs) were applied to identify regions with significant differences compared to control trials. These models were also used to evaluate the impact of perturbation on muscle activation MAV relative to baseline, accounting for inter-subject variability. Additionally, LMMs were used to compare the onset timing of muscle activation across different muscles. Specifically, comparisons between the onset timings of proximal muscles were exclusively made against those of distal muscles for each leg, with no mutual comparisons between different muscle groups or legs. Across all statistical analyses performed, a significance level of α=0.05 was utilized, with the false discovery rate (FDR) method (Benjamini and Hochberg, 1995) applied for multiple comparison correction. Statistical analysis was done using Matlab R2020b (MathWorks Inc., Natick, United States).

## 3 Results

### 3.1 AMP performance, body-weight support, and mechanical response

We evaluated AMP’s perturbation generation and participants’ mechanical responses across different timings and directions. AMP produced frontal-plane moment pulses averaging 47.7 N m for rightward and 50.4 N m for leftward perturbations (Supplementary Figure S4). These pulses induced maximal frontal-plane trunk rotations of 8.5deg and −12.8deg in the rightward and leftward directions, respectively, compared to trunk posture in the control trials (Supplementary Figure S3). Notably, upper-body roll rotation contrasted with only a minor 2.5 cm CoM shift in the ML direction (Supplementary Figure S5). The utilization of an active overground bodyweight support system as a safety precaution, along with the carried mass of the AMP, each contributed to minor alterations in trunk and hip posture (notably forward-leaning to counterbalance the backpack-like AMP) and leg kinematics (Supplementary Figures S10–14). These effects remained consistent across all perturbation conditions. Further details of the perturbation characteristics and participant mechanical response can be found in the Supplementary Materials.

### 3.2 Muscle activation patterns in response to moment perturbations

**FIGURE 2:**
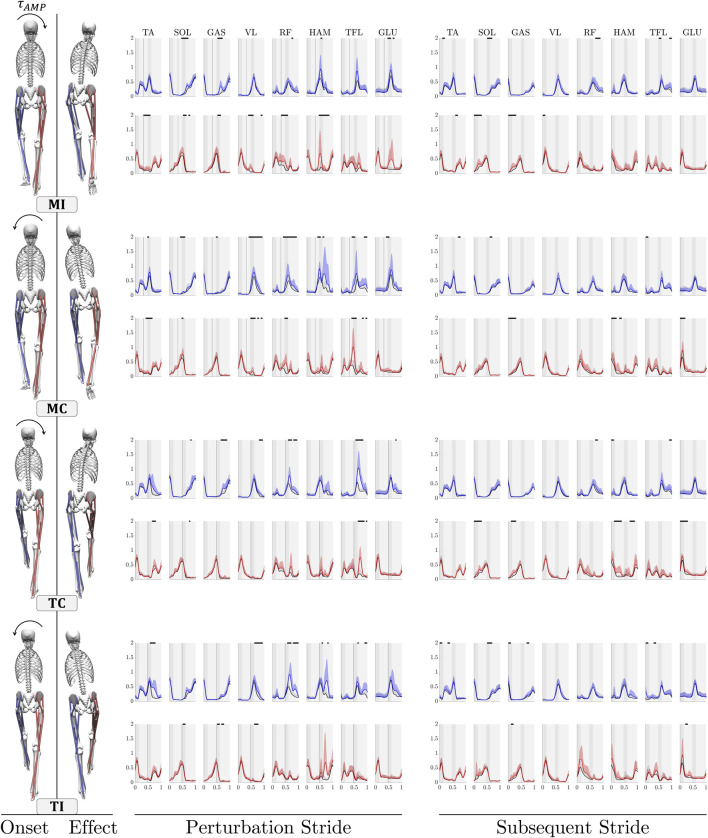
Comparison of the grand mean EMG activity in eight lower limb muscles during upper-body gyroscopic moment perturbations. Each figure compares the mean + SD of EMG across all participants for both control trials (black) and perturbed trials (red for the right leg and blue for the left leg) within a single stride. Each figure begins with the right leg making contact with the ground, and the shaded regions in the background indicate the phases of single (light grey) and double support (dark grey). Regions showing statistically significant differences between the perturbed and unperturbed experimental conditions are denoted by distinct piece-wise horizontal black lines. These differences are determined at a significance level of α=0.05 (FDR-corrected). Each pair of stick figures visually demonstrates a perturbation case, with the first figure representing the moment of perturbation application and the second figure illustrating the resulting perturbation effect. *MI*: Midstance-Ipsilateral, *MC*: Midstance-Contralateral, *TC*: Touchdown-Contralateral, *TI*: Touchdown-Ipsilateral.

We compared the ensemble average of muscles’ activation in perturbed trials with those of control trials to analyze the changes in muscle activity patterns in response to upper-body gyroscopic moment perturbations (Figure 2). The EMG activity of the right and left leg is visually represented by the colors red and blue, respectively, while the color black signifies measurements from control trials in the figure. In the following, we outline the responses, beginning with the left leg reaction, followed by the reactions of the right leg. This sequence is not indicative of the significance of each leg’s response.

**MI** *(rightward perturbation at right leg mid-stance)*: Ipsilateral perturbations at mid-stance result in distinct changes in activation patterns of left proximal hip-extensor muscles, HAM and GLU, during the double support phase, accompanied by increased activation in RF in the next single support phase. Additionally, minor increases in activation are noted in the plantar-flexor muscles SOL and GAS. On the right side, the perturbation triggers the initiation of long-latency responses in the proximal and distal muscles. Increased activity is observed in proximal biarticular thigh RF and HAM muscles, as well as the knee-extensor VL, during the stance and double support phase. For distal muscles, increased activity changes are noted for plantar-flexor SOL and biarticular GAS. In the subsequent stride following the perturbation, comparatively lower increases are observed for the left proximal muscles. No other significant changes are observed, except for some minor increases in the activity of right SOL and GAS muscles.

**MC** *(leftward perturbation at right leg mid-stance)*: When contralateral perturbations occur during the mid-stance, the response is characterized by notable increases in activity of the proximal muscles surrounding the hip joint, such as RF, HAM, TFL, and GLU, with HAM and TFL demonstrating higher levels of variability. The VL muscle at the knee joint also demonstrates significantly heightened activities. In the right leg, the primary response is characterized by a rapid and pronounced increase in the activity of the right leg’s TFL and RF throughout the stance phase. Upon analyzing the next stride after perturbation, no significant changes are observed in muscle activity, except for minor initial increases in the proximal right leg HAM and GLU muscles.

**TC** *(rightward perturbation at left leg touchdown)*: When contralateral perturbations are applied during the left leg’s touchdown, a rapid and substantial response is generated by the left leg’s TFL, followed by the RF muscle. In the distal muscles, increased activity is observed in the GAS, along with minor changes in the VL muscle. On the right leg, pronounced increased activity in the TFL during the swing phase ensures proper leg adjustment. Among the distal muscles, only the TA shows a slight increased activity during the swing phase, assisting in foot clearance. In the subsequent stride after the perturbation, the right leg’s HAM and GLU muscle and ankle plantar flexors (SOL and GAS) exhibit some activity, likely due to a higher level of leg loading.

**TI** *(leftward perturbation at left leg touchdown)*: In response to ipsilateral perturbations during the left leg’s touchdown, increased muscle activities are primarily observed in the proximal muscles of the left leg, including co-contraction of the RF and HAM, along with changes in the TFL during the late stance. Additionally, increased activity is noted in the VL. Noteworthy changes are also observed in the TA muscle at the ankle joint among the distal muscles of the left leg. On the right leg, only the HAM muscle exhibits increased activity with high variations during the swing phase. In the subsequent stride after the perturbation, the biarticular muscles of the right leg show increased activity; however, this change is not statistically significant.

### 3.3 Relative muscle recruitment in perturbation recovery

**FIGURE 3:**
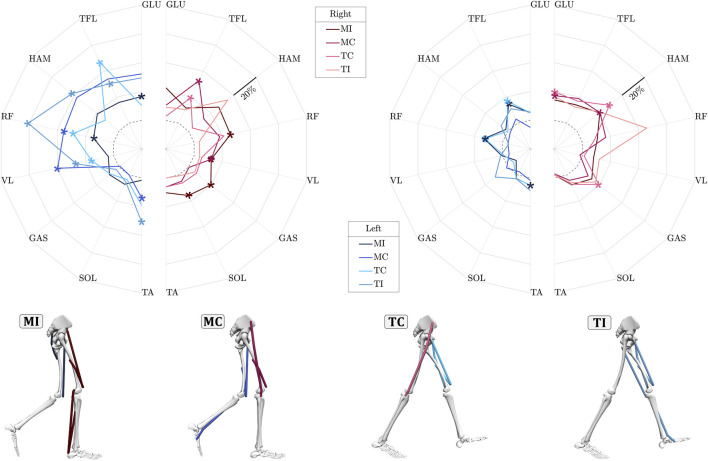
Relative mean absolute value (MAV) of eight lower-limb muscles activation in response to upper-body gyroscopic perturbations for the perturbation stride (left plot) and the subsequent stride (right plot). The relative mean is calculated as the normalized MAV difference between perturbed trials and control trials in percent. The dashed circle in these graphs indicates the zero value, meaning no difference in MAV of muscles in perturbed cases w.r.t. those of unperturbed were detected. Statistically significant changes in individual muscle MAV (FDR-corrected, α=0.05 ) are marked with asterisks (*). The stick figures at the bottom illustrate the muscles with significant MAV change during the perturbation stride across all experimental conditions. *MI*: Midstance-Ipsilateral, *MC*: Midstance-Contralateral, *TC*: Touchdown-Contralateral, *TI*: Touchdown-Ipsilateral.

We analyzed the relative mean absolute values (MAV) of each of the measured muscle activities in one stride, comparing them to their respective base values in control trials to assess the muscles’ involvement in coping with perturbations (Figure 3). During the perturbation stride, nearly all muscles exhibited increased activities in response to the four types of perturbations in both legs, except for the SOL and GAS muscles in the *MC* experimental case, which showed no difference in activity. Notably, the SOL and GAS muscles consistently displayed relatively low relative MAV values, reaching a maximum of 8% compared to other muscles, particularly in the left leg. Comparing the relative MAV of the perturbation stride to the subsequent stride, it is evident that the majority of heightened muscle activities occur during the perturbation stride, particularly in the left leg. Upon individual analysis of the muscles during the perturbation stride, the biarticular muscle RF stood out as particularly important among the studied muscles (Hypothesis 1 & 2). This muscle consistently exhibited statistically higher responses during the perturbation stride for the left leg (Supplementary Table S1). Following closely, the VL of the left leg exhibited the next most consistent statistical changes during the perturbation stride, showing significantly higher MAV changes in all cases except the *MI* case. In almost all cases, either RF or HAM ranked among the top three muscles with the strongest response (Hypothesis 2), though statistical analysis revealed significant changes only for HAM in the *TI* case during the perturbation stride. Next in line is the TFL muscle, notable for maintaining relatively consistent activation levels across different perturbation cases and legs, with the exception of the *MI* case in the left leg.

### 3.4 Timing of muscle activation

**FIGURE 4:**
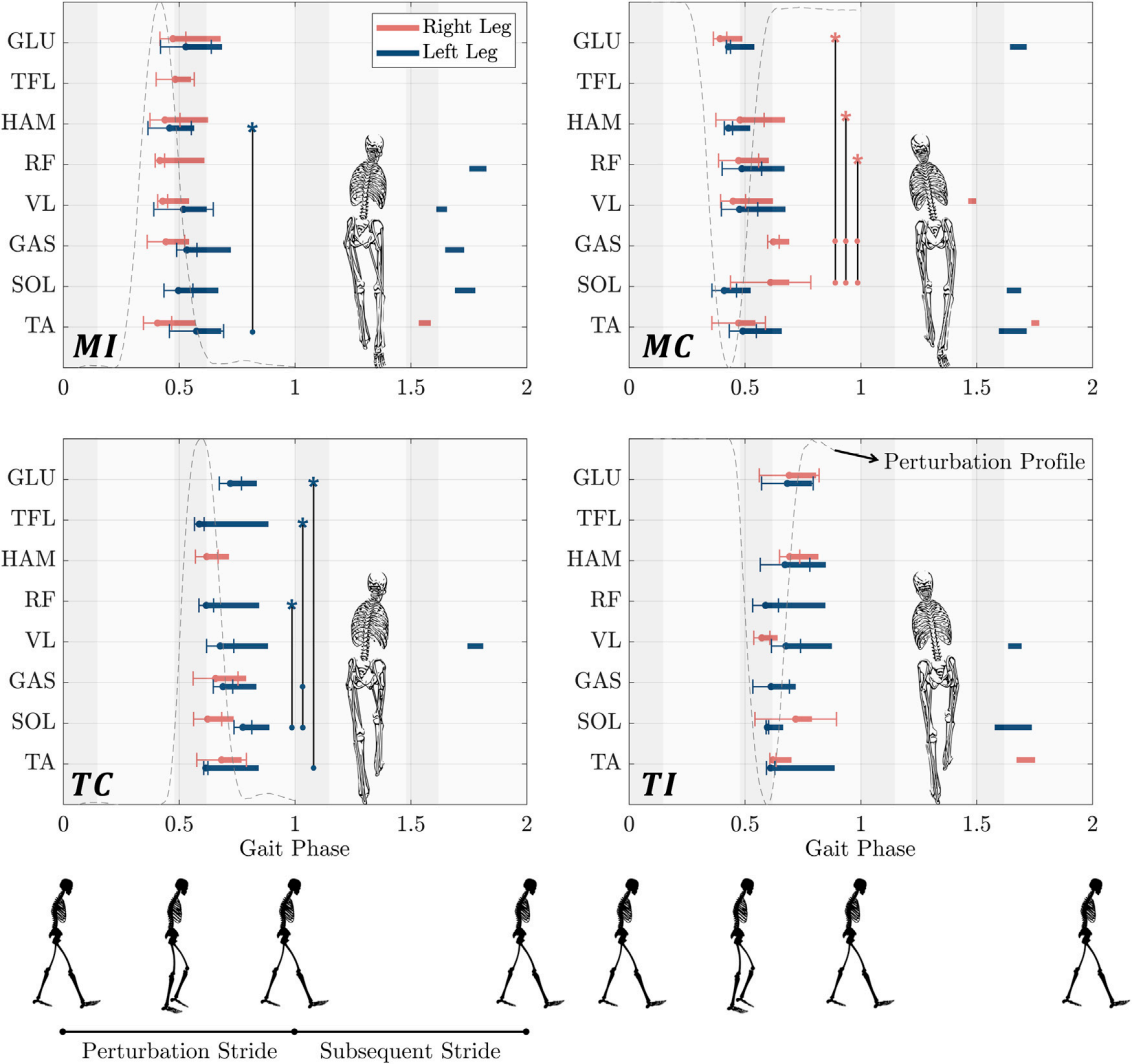
Time-synchronized organization of the excitatory responses of eight lower limb muscles in response to upper-body gyroscopic perturbations at right midstance (first row) and left touch down (second row). The horizontal bars represent an increase in muscle activity compared to the unperturbed control trials that exceeded one SD above the ensemble average of control trials and lasted for a minimum of 30 ms. The beginning of each bar is marked by a line denoting the standard deviation. Bar endings were defined as the point when perturbed trial muscle activity dropped one SD below the average of control trials. The muscle excitatory responses for the right and left leg are shown in red and blue, respectively. Vertical lines with star symbols represent statistical differences in specific muscle activation timings. Stars indicate reference data points, while circle markers represent comparison points. Statistical analysis was performed using a linear mixed-effects model with a significance level of α=0.05 (FDR-corrected). The shaded regions in the background indicate the phases of single (light grey) and double support (dark grey). The stick figures illustrated at the bottom indicate the phases of a gait cycle, assuming that a stride duration is one. *MI*: Midstance-Ipsilateral, *MC*: Midstance-Contralateral, *TC*: Touchdown-Contralateral, *TI*: Touchdown-Ipsilateral.

In Figure 4, we compared muscles’ response timing in response to perturbations to determine if proximal muscles react faster than distal muscles to cope with the perturbations (Hypothesis 3). The muscle’s excitatory response to the gyroscopic perturbation was identified by observing a significant increase in muscle activity that exceeded one standard deviation beyond the grand average observed in control trials (Tang et al., 1998).

**MI** *(rightward perturbation at right leg mid-stance)*: In the case of the *MI* perturbation, the left leg’s biarticular HAM exhibited the fastest response among all the measured muscles, with the response time of 232 ms. This reaction time was observed to be statistically significantly faster than that of TA (p=0.0369) . On the right leg, while the RF and TA are among the muscles measured, no statistically significant difference was observed in the onset latency of these muscles (Supplementary Table. SII and SIII).

**MC** *(leftward perturbation at right leg mid-stance)*: In response to the *MC* perturbation, while no statistically significant difference was observed in the onset latency of left leg muscles, the proximal muscles, GLU, and the biarticular RF and HAM, exhibited significantly faster responses than SOL and GAS on the right leg. Among the proximal muscles on the right leg, GLU showed the quickest response (166 ms), statistically faster than SOL (385 ms, p=0.0057 ) and GAS (396 ms, p=0.0057 ). The biarticular RF and HAM displayed similar latencies (247 ms and 252 ms, respectively) and were both statistically faster than SOL and GAS (p=0.0391) . No other statistically significant differences were observed.

**TC** *(rightward perturbation at left leg touchdown)*: In this case, TFL demonstrated the quickest response among the proximal muscles of the left leg (160 ms). Compared to the distal muscles, TFL exhibited a statistically significantly faster response than SOL (349 ms, p=0.0005 ) and GAS (263 ms, p=0.0323 ). Additionally, the left RF was statistically faster than the distal SOL muscle (p=0.001) . However, GLU was observed to have a slower onset latency than TA muscle (p=0.0323) . Regarding the muscles on the right leg, although the proximal HAM exhibited a faster onset latency than the distal muscles, this difference was not statistically significant. No other statistically significant differences were observed.

**TI** *(leftward perturbation at left leg touchdown)*: In the case of the *TI* perturbation, the left biarticular RF displayed the fastest response among all the leg muscles (162 ms). On the right leg, VL exhibited the fastest response at 146 ms, followed by the biarticular proximal RF muscle (173 ms). However, no statistically significant differences were observed among these muscles or any others.

## 4 Discussion

This study aimed to examine the muscle recruitment patterns involved in reactive balance adjustments during laterally perturbed human walking, with a specific focus on the involvement of proximal and distal postural muscle activity and their timing of activation. This work stands out from previous research by applying angular momentum perturbations to the upper body in the frontal plane via AMP, deviating from conventional force-type or whole-body pitch angle perturbations (van Mierlo et al., 2022; Hof A. L. and Duysens J., 2018; Vlutters et al., 2018; van Mierlo et al., 2023). Despite the fact that the perturbations produced by the AMP do not replicate real-world scenarios encountered in daily life, our objective with the AMP was to construct an artificial setting aimed specifically at isolating the rotational component of human balance. This approach allows for a deeper understanding of the neurological mechanisms underlying balance control in the frontal plane during gait, providing insights that may not be easily attainable through conventional perturbation methods.

Our findings confirm several hypotheses: They support our first hypothesis, indicating that muscle responses to perturbations are predominantly mediated by proximal muscles across various perturbation scenarios. Additionally, our results validate the second hypothesis, suggesting that biarticular muscles exhibit significant involvement in counteracting balance threats. Importantly, these results exhibit generalizability across different perturbation cases. Furthermore, our findings partially support the third hypothesis by showing that, in cases of statistical significance, the fastest muscle responses typically originate from muscles closest to the perturbation site. However, we observed that this organization is contingent on the timing and direction of the perturbation. We also observed variability in the magnitude of EMG activity among individual muscles, dependent on the specific instance and direction of perturbation. Lastly, our findings reveal that corrective responses to upper-body moment perturbations typically occur within the same stride as the perturbation. These findings hold promise for generalization to other perturbation cases and offer significant potential to inform the development of assistive devices and clinical interventions, particularly benefiting elderly individuals.

### 4.1 Primary involvement of proximal muscles in perturbation recovery

The muscle activation patterns in Figure 2 suggest that the reactive responses of the muscles are adaptively tailored to match the specific type of perturbation, rather than being globally applied regardless of perturbation context. However, as postulated in this study (Hypothesis 1), one notable trend observed across all perturbation cases is the prominent use of a hip strategy as the primary mechanism for restoring balance. Given that the perturbations primarily affect the trunk and pelvis, the hip strategy exhibits crucial involvement in the recovery process. While there were instances where an ankle strategy was also employed, it was not as prevalent. This result is consistent with prior research highlighting the significance of the hip strategy and indicating a limited involvement for the ankle strategy (Rietdyk et al., 1999; Best et al., 2019; van Mierlo et al., 2022). This discrepancy in strategy prevalence is linked, in part, to the substantial perturbation torque applied by the AMP, reaching approximately 50 Nm (Supplementary Figure S4). Prior studies suggest that, in milder perturbations, stepping and ankle strategies would have been more commonly employed, as they are the primary balance strategies during walking (Hof et al., 2010b). The ankle strategy primarily facilitates minor adjustments in postural sway to regain balance, while the hip strategy relies on the musculature of the hip joint and proximal muscles to effectively counteract more substantial perturbations (Horak and Nashner, 1986). The hip strategy can even come into play when the ankle strategy proves inadequate (e.g., during ankle inversion) to address the lateral sway (Horak and Nashner, 1986; Stanek et al., 2011). Accordingly, our results further emphasize the critical involvement of proximal muscles in maintaining balance and suggest that the neuromuscular system prioritizes large-scale postural adjustments over finer, more localized adjustments when dealing with substantial perturbations.

### 4.2 Notable involvement of biarticular muscles in perturbation rejection

Analysis of the MAV (Figure 3) in response to upper-body gyroscopic perturbations revealed that in all four perturbation conditions, the biarticular RF showed statistically significant higher levels of muscle activity compared to the control trials during the perturbation stride. This observation supports our second hypothesis that thigh biarticular muscles exhibit critical involvement in generating reactive balance adjustments. A key reason for this notable involvement in the perturbations studied may lie in the unique mechanical coupling of these two-joint muscles, which not only enables more efficient energy transfer between the hip and knee but may also reduce neural control complexity by allowing a single muscle to coordinate two joints (Schumacher et al., 2020). While the RF primarily acts as a hip flexor and knee extensor in the sagittal plane, its biarticular anatomy enables indirect contributions to frontal-plane stability. For instance, by stabilizing knee extension during weight acceptance, the RF may enhance limb stiffness, reducing mediolateral trunk displacement caused by AMP-induced angular momentum. Simultaneously, its hip flexion action could adjust trunk-pelvis alignment, indirectly aiding in counteracting roll perturbations.

The importance of biarticular muscles has been extensively investigated in prior research, demonstrating their critical involvement in efficient (Junius et al., 2017; Mohseni et al., 2022) and robust (Dean and Kuo, 2009) locomotion. These two-joint muscles coordinate joint movements, transfer energy, and prevent joint overextension by mechanically coupling adjacent joints (Schumacher et al., 2020). Our findings extend this understanding to frontal-plane perturbations during gait: the heightened RF activity suggests that sagittal-plane biarticular muscles contribute to multiplane stability by modulating limb mechanics (e.g., knee stiffness) that secondarily influence frontal-plane balance. The biarticular muscles also exhibit a dominant involvement in rotational motions during the balance and swing phases of locomotion (Schumacher et al., 2020) and help synchronize individual locomotor subfunctions (Schenau, 1989; van Ingen Schenau et al., 1987; Nilsson et al., 1985). Their capacity to exert substantial force perpendicular to the leg axis (Hof, 2001) makes them highly effective at managing angular momentum and preserving postural balance. In our study, the RF’s force transmission across the hip and knee may create a stabilizing “kinetic chain,” redistributing AMP-induced destabilizing forces along the leg to mitigate trunk roll. Simulations and robotic studies have also proved that biarticular structures, can stabilize the trunk during walking and generate appropriate leg swing motions (Lakatos et al., 2014; 2016; Sharbafi et al., 2017; Dean and Kuo, 2009). Our results align with these models but uniquely demonstrate that even sagittal-plane biarticular muscles (e.g., RF) play a critical role in managing frontal-plane perturbations, likely through their mechanical coupling of joints rather than direct frontal-plane torque production.

Perturbation studies further highlight their importance. For instance, humans exhibit rapid HAM activity in response to angular momentum perturbations during stumbling (Pijnappels et al., 2005). The biarticular thigh muscles, RF and HAM, are strongly involved in counteracting upper-body pitch perturbations during both standing and walking (Schumacher et al., 2019; van Mierlo et al., 2022). This supports a hierarchical control strategy where biarticular muscles act as “mechanical shortcuts” to simplify multi-joint coordination under time-critical balance challenges, thereby enabling rapid, whole-limb adjustments to counteract AMP-induced angular momentum. Additionally, biarticular muscles influence frontal plane balance. During the late stance phase of walking, the GAS muscle helps rotate the body towards the contralateral leg, aiding in the generation of frontal plane angular momentum (Neptune and McGowan, 2016). These findings from prior works, along with the results from this study, provide additional evidence supporting our hypothesis that biarticular muscles exhibit strong involvement in recovery from perturbations.

### 4.3 Proximal muscles react faster, but in no proximal-to-distal sequence

In our study, we hypothesized that the muscle reactive response would be initially and predominantly concentrated at the site of the perturbation, then cascade in a proximal-to-distal sequence to further augment the initial response and compensate for any postural misalignment incurred therein (Hypothesis 3). This hypothesis proposes that proximal muscles respond more swiftly than distal muscles due to their closer proximity to the perturbation point, enabling them to detect perturbations earlier and initiate compensatory muscle activations more rapidly. This is attributed to the presence of proprioceptive receptors within the proximal muscles and nearby joints that can effectively detect the perturbation signal, promoting prompt postural adjustments.

Our analysis of muscle timing sequences (Figure 4) revealed, a consistent pattern across all perturbation cases: proximal muscles were the initial recruits to counteract perturbations whenever there was a statistically significant difference in onset latency among muscles. In the *MI* case, the left biarticular HAM exhibited the fastest response. Notably, although the right TA appeared to activate before the right leg’s proximal muscles, this difference was not statistically significant. In the *MC* case, the right GLU was the quickest to activate and significantly faster than the distal muscles SOL and GAS. In the *TC* case, our observations indicated that the proximal left TFL exhibited the fastest response, which was statistically significant compared to the distal muscles SOL and GAS. Finally, in the *TI* case, no statistically significant differences in onset latency were found among muscles for both legs.

Previous investigations into the organization of excitatory postural responses in muscles following perturbations have yielded diverse outcomes. Horak et al. demonstrated that during standing, muscle activity in response to altered support-surface configurations initiates in ankle joint muscles and subsequently propagates to thigh and trunk muscles in a sequential manner (Horak and Nashner, 1986). However, this sequential activation pattern was found to be reversed on a short support surface. A similar sequence reversal was also observed in the work of Woollacott et al. with older adults when the direction of the standing platform perturbation was changed from forward to backward (Woollacott et al., 1986). These earlier findings were partially supported by the work of Moore et al., where they identified a sequence of muscle activations but noted that the initial burst of abdominal muscle activity precedes the onset of activity in distal muscles. They further explored the effect of perturbation direction on the organization of automatic postural responses and found that the temporal order of muscle activation can be modulated by the direction of the perturbation (Moore et al., 1988). More recently, in a study investigating unexpected waist-pull perturbations in both AP and ML directions, the ankle muscles were found to exhibit the highest rate and magnitude of activation (Zhu et al., 2022). Regarding walking with translational platform perturbations, the response generated by distal muscles (TA) was found to occur prior to the changes in proximal muscles (Tang et al., 1998). However, it was reported that this sequential pattern varied across trials. Similarly, for pelvis perturbations in the ML direction during slow walking, it was found that the gluteus medius exhibited the earliest response onset, followed closely by other distal muscles like TA (Vlutters et al., 2018). The current findings support the hypothesis that proximal muscles, in close proximity to our AMP perturbation source, are actively involved in the initial response to upper-body moment perturbations. However, these results challenge the hypothesis that automatic muscle responses follow a specific sequential pattern in walking. This aligns with previous works and suggests that the composition of reactive muscle responses is a complex process influenced by multiple factors such as timing, direction (Woollacott et al., 1986; Moore et al., 1988), and intensity (Manchester et al., 1989).

### 4.4 Enhanced muscle activation in the support leg

In response to ML perturbations, excluding the *MI* case, the left leg demonstrated greater overall proximal muscle activations compared to the right leg during the perturbation stride (Figure 3). Furthermore, in experimental scenarios where the body was perturbed towards the left leg (i.e., *MC* and *TI* cases), proximal muscles exhibited even higher activation levels, except for the TFL muscle in the *TC* case. A probable explanation behind higher muscle activations of the left leg could be that perturbation recovery during walking is impacted by the duration of the perturbation pulses. According to the perturbation profile generated by the AMP (Supplementary Figure S4), perturbation effects extend into the subsequent step. Across all experimental conditions, the left leg remains in or transitions to stance, amplifying its involvement in addressing the perturbation. Another less likely factor that could contribute is related to the participants’ awareness of perturbation timing (Major et al., 2020). Despite the random initiation of perturbations, the participants might have developed the ability to predict these occurrences as they traversed the initial force plate. This anticipation could have led to preparatory responses (Aruin et al., 1998), consequently intensifying the involvement of the upcoming stance leg in executing corrective maneuvers.

### 4.5 Methodological limitations

#### 4.5.1 Gyroscopic perturbation generation

The AMP utilized in this study is equipped with a single CMG, which poses a limitation on the independent manipulation of gyroscopic moment direction. As the gimbal rotates, the gyroscopic torque vector follows, causing not only a roll perturbation but also introducing a pitch component in the sagittal plane. To mitigate this issue, in this study, the gimbal angle was restricted, allowing for approximate projection of the gyroscopic moment in the intended direction during normal operation. Consequently, the maximum achieved torque in the frontal plane was measured at 49 N m (averaging both directions). However, pitch torques were still present. All perturbations produced a maximum backward pitch moment of 13.3 N m followed by a maximum forward pitch moment of 8.4 N m (Supplementary Figure S4). Notably, despite these unwanted pitch torques arising from the underactuated nature of the AMP system, analysis from our previous work on the same dataset (Mohseni et al., 2024b) suggests that they did not require substantial compensatory adjustments from participants. To counteract the unwanted pitch torques, a scissored-pair CMG can be employed, allowing non-aligned torques to cancel each other (Meijneke et al., 2021).

#### 4.5.2 Non-uniform overground walking

In this study, participants were instructed to initiate walking from a standing position, take about three to four strides at their self-selected speed, and then come to a stop at the end of the walkway. A limitation of this non-uniform overground walking approach is the constrained length of the available walkway. While previous studies have demonstrated the similarity between overground walking and treadmill walking in terms of kinematics and stepping responses to perturbations (Riley et al., 2007; Zadravec et al., 2017), the use of a treadmill allows for better control of experimental conditions, more repetitions within a given timeframe, and improved measurement consistency (Zadravec et al., 2017). Nevertheless, treadmill walking is associated with less variability in gait patterns (Hollman et al., 2016; Yao et al., 2019) and can induce changes in joint moments and EMG activity of specific muscles (Lee and Hidler, 2008; Yao et al., 2019). Therefore, a more effective approach may involve utilizing an extended overground walkway to address the limitations associated with the constrained length of the walkway.

Another important factor to consider is the self-selected walking speed, which can vary among participants and may influence EMG patterns and balance strategies. As walking speed increases, preferred balance strategies shift from being in-stance to stepping strategies (Matjačić et al., 2019). However, it is challenging to interpret how slowing down at the end of the walkway in our study may have affected balance or EMG activities (Hagedoorn et al., 2022).

#### 4.5.3 Effects of perturbation setup on nominal gait

One notable consequence of utilizing the bodyweight support system, combined with the addition of the AMP, was trunk flexion; −3.5deg and −7.8deg in the ‘BWS’, and ‘BWS+ $\bar{AMP}$ ’ experimental cases. The forward tilt of the trunk results in a shift of the body’s CoM, thereby influencing muscle activation patterns and joint moments. Even small changes in trunk flexion, up to 10deg, have been observed to increase hip and ankle moments and heighten the activity of the HAM and GAS muscles. Furthermore, the activity of knee medial and lateral flexor muscles demonstrates a strong correlation with trunk flexion (Preece and Alghamdi, 2021; Alghamdi and Preece, 2020). One potential solution to mitigate these undesired effects could be using a pint-sized CMG, which weighs 1.2 kg and has a more streamlined design (Meijneke et al., 2021).

Furthermore, it’s imperative to acknowledge that responses to external perturbations can be influenced by various factors, including the intensity (O’Connell et al., 2016; Hof A. and Duysens J., 2018) and onset timing of the perturbation (Golyski et al., 2022; Hof A. and Duysens J., 2018; Schillings et al., 2000). In our study, we utilized the AMP in only one intensity level and chose two specific timing points during the gait for perturbation application. The latter choice was influenced by technological constraints in our experimental apparatus for discerning distinct gait phases. Moving forward, it is essential to explore different intensity levels and evaluate the impact of different perturbation timings (Golyski et al., 2022; Darici et al., 2020) to ascertain the generalizability of our findings to broader contexts.

#### 4.5.4 Potential for additional EMG measurements

In this study, among the eight lower-limb muscles recorded, three muscles are directly involved in movements in the frontal plane: GLU and TFL around the hip, and TA around the ankle joint. While movements primarily occur in the sagittal plane at the knee joint, there is potential for additional EMG measurements around the hip and ankle joints, such as those for the gluteus medius and peroneus longus, in future studies. However, it’s essential to acknowledge that not all muscles can be effectively measured using surface EMG sensors, suggesting room for complementary approaches like force-based sensing (Schäfer et al., 2024).

## 5 Conclusion

This study investigated the interplay of coordinated muscle actions in addressing upper-body gyroscopic moment perturbations and maintaining balance during human overground walking. By employing an angular momentum perturbator to apply targeted frontal-plane torque perturbations, we identified the prominent involvement of proximal muscle activation. Notably, the biarticular rectus femoris proved to be instrumental in shaping reactive responses across various scenarios. Our findings also indicated that the primary corrective muscle actions typically occur within the same stride as the perturbation. Additionally, in instances where statistical significance was found, the quickest responses typically originated from proximal muscles, which are in close proximity to the perturbation site. However, muscle recruitment for coping with perturbations did not strictly adhere to a proximal-to-distal sequence. The temporal sequence and muscle activation magnitude were found to be modulated by the timing and direction of the perturbation. These insights—particularly highlighting the prominence of proximal muscle activation (e.g., the biarticular Rectus Femoris) and phase-specific recruitment patterns in response to trunk-level perturbations—inform the design and control of assistive or rehabilitation devices by emphasizing: (1) targeted support or actuation around the hip and upper-leg region with biarticular architectures (Davoodi et al., 2024; Sharbafi et al., 2018), where the largest compensatory torques occur; (2) integration of real-time, phase-adaptive controllers that respond to specific gait events and perturbation direction; and (3) refined musculoskeletal models that incorporate the observed reflex timing and magnitude to personalize assistance, especially for older adults or those with neuromuscular impairments.

## Data Availability

The datasets presented in this study can be found in online repositories. The names of the repository/repositories and accession number(s) can be found in the article/Supplementary Material.
