# Supplementary material for: Muscular responses to upper body mediolateral angular momentum perturbations during overground walking
Source: Front Bioeng Biotechnol. 2025 Apr 11;13:1509090. doi: 10.3389/fbioe.2025.1509090 (PMC12021904; doi:10.3389/fbioe.2025.1509090)
Supplement: Supplementary file 1 [file DataSheet1.pdf]

Supplementary Materials

1 METHODS

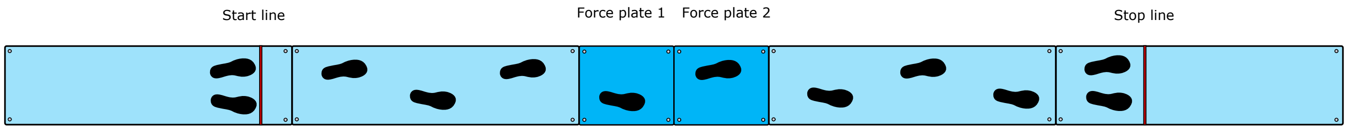

Figure S1: Diagram of the instrumented walkway used in the experiment. From standing, each participant initiated overground walking with their left leg and walked 3 steps along a raised platform before contacting the first and second force plates (middle panels; the same color as the walkway but highlighted here for clarity) on the 4th and 5th steps (right and left steps, respectively), and continued for another 3-4 steps before stopping. The position of the start line was adjusted for each participant to ensure the correct foot would fall completely within the surface of the force plates.

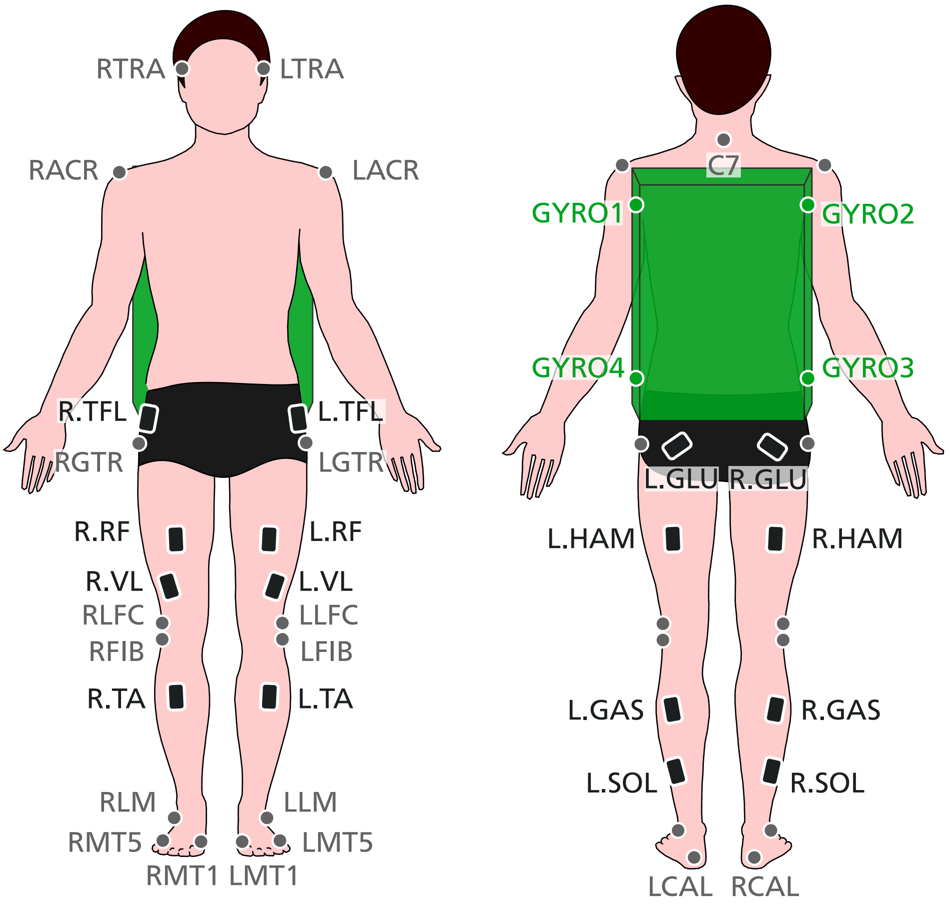

Figure S2: Locations and names of reflective motion capture markers on the body (grey circles) and AMP (green circles), and EMG sensors (black rectangles) Schumacher et al. (2019).

2 RESULTS

AMP generated consistent and dynamic torque perturbations.

We checked for the performance of the AMP in generating perturbation profiles at different timings and directions to verify that the output of the AMP aligned with our desired profile. We calculated the mean and standard deviation of gyroscopic torque, based on measured gimbal angular velocity, across all the trials and participants; see Fig. S4. Despite the realized peak torque falling slightly below the target of $60\,\mathrm{N\,m}$ (mean $47.7\,\mathrm{N\,m}$ for rightward perturbations and mean $50.4\,\mathrm{N\,m}$ for leftward perturbations), the rise and fall dynamics were accurately tracked. Furthermore, the small standard deviation observed in the measured profile confirmed the consistent generation of perturbations throughout multiple trials.

AMP induced specific upper-body roll perturbations.

To examine the resulting body posture of the participants, we assessed the upper-body roll (rotation in the frontal plane) deflections, the whole-body CoM, and joint angles. After the application of the torque perturbation, the trunk exhibited a maximum rotation up to $8.5\,\mathrm{deg}$ and $-12.8\,\mathrm{deg}$ for respectively rightward and leftward AMP torques with respect to the trunk posture in the control trials in the absence of perturbation. The peak roll torque reached approximately $170\,\mathrm{ms}$ to $270\,\mathrm{ms}$ after the onset of the perturbation (Fig. S4). In contrast to the significant upper-body roll rotation, the CoM only shifted by an average of $2.5\,\mathrm{cm}$ in the ML direction; see Fig. S5. The participants' mechanical response following the perturbation onset resulted in $7.1\,\mathrm{deg}$ deflection in the pelvis for rightward perturbation cases (*MI* and *TC*), and an $-8.7\,\mathrm{deg}$ deflection for leftward perturbation cases (*MC* and *TI*); see Fig. S6. In terms of the lower-limb joints: The right hip abduction/adduction reached a peak deviation of 4.6 to $8.5\,\mathrm{deg}$, while the left hip deviation ranged from 3.8 to $8.4\,\mathrm{deg}$ in comparison to the hip angles observed during the control trials. The right knee flexion/extension changed about 4.3 to $5.8\,\mathrm{deg}$, whereas the left knee showed a range of 2.1 to $10.9\,\mathrm{deg}$. The right ankle inversion/eversion changed around 4.5 to $5.7\,\mathrm{deg}$, and the left ankle exhibited a range of 2.3 to $4.9\,\mathrm{deg}$; see Figs. S7–S9.

Influence of AMP and bodyweight support

Next, we tested the extent to which the body weight support system RYSEN and the weight of the AMP influenced the gait kinematics and GRF during overground walking. The addition of the RYSEN system led to a more forward-bent trunk ($-3.5\,\mathrm{deg}$, $p = 0.001$), a slightly twisted pelvis ($0.9\,\mathrm{deg}$, $p = 0.004$), and increased left hip abduction ($1.5\,\mathrm{deg}$, $p < 0.001$) with a left hip medial rotation ($1.5\,\mathrm{deg}$, $p = 0.01$). No difference was found in the right hip, knee, and ankle joints; see Figs. S10–S14. By comparing the GRF data of the 'Free' and 'BWS' conditions, no change in the force magnitude was identified (see Fig. S16). Wearing the AMP resulted in an even more forward-bent trunk ($-7.8\,\mathrm{deg}$, $p < 0.001$), a slight lateral trunk inclination ($0.9\,\mathrm{deg}$, $p < 0.001$), and a trunk twist ($1.5\,\mathrm{deg}$, $p < 0.001$). It also altered the pelvis angle ($2.8\,\mathrm{deg}$, $p < 0.01$) in the sagittal plane, and ($1.1\,\mathrm{deg}$, $p < 0.004$) in the horizontal plane. In the lower-limb joints, the right hip demonstrated adduction ($0.6\,\mathrm{deg}$, $p = 0.002$), flexion ($2.9\,\mathrm{deg}$, $p = 0.002$), and inward rotation ($3.2\,\mathrm{deg}$, $p < 0.001$). The left hip also exhibited adduction ($1.4\,\mathrm{deg}$, $p < 0.001$)) and rotation ($1.3\,\mathrm{deg}$, $p = 0.002$). Additionally, there was an increase in knee flexion ($1.6\,\mathrm{deg}$, $p < 0.001$). Finally, both the right and left subtalar joints showed lateral movement, averaging ($-1.6\,\mathrm{deg}$, $p < 0.001$) and ($-1.8\,\mathrm{deg}$, $p = 0.002$), respectively. Comparing GRF data between the 'Free' and 'BWS+$\overline{\text{AMP}}$' conditions showed an average increase of ($124\,\mathrm{N}$, $p = 0.008$) in the vertical direction. No other significant differences in the joint kinematics or GRF were found.

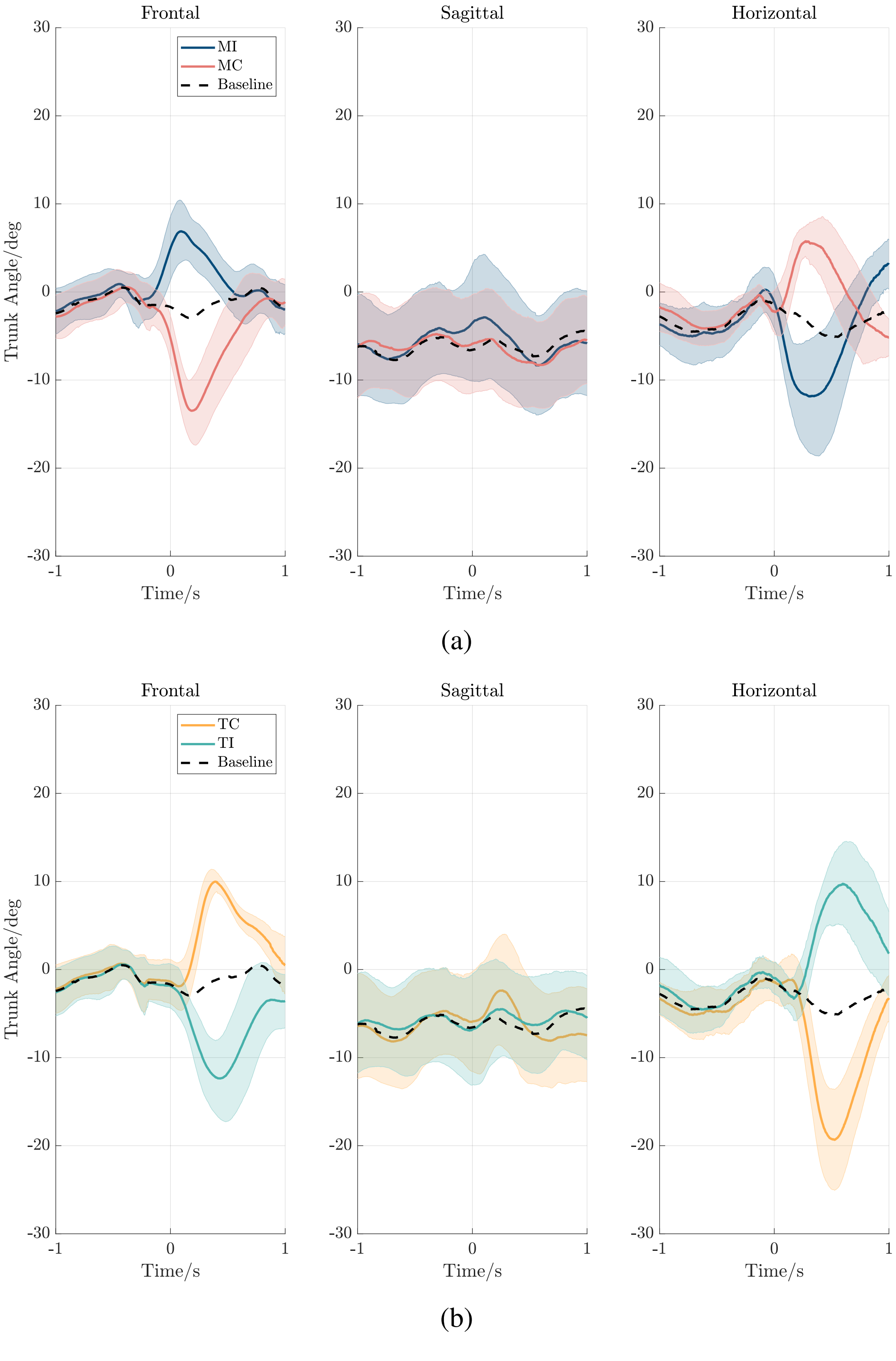

Figure S3: Grand mean±SD of trunk angle for all four perturbation cases across 10 participants in frontal, sagittal, and horizontal planes. Time 0 indicates the moment when the left leg touches the second force plate. *MI*: Midstance-Ipsilateral, *MC*: Midstance-Contralateral, *TC*: Touchdown-Contralateral, *TI*: Touchdown-Ipsilateral.

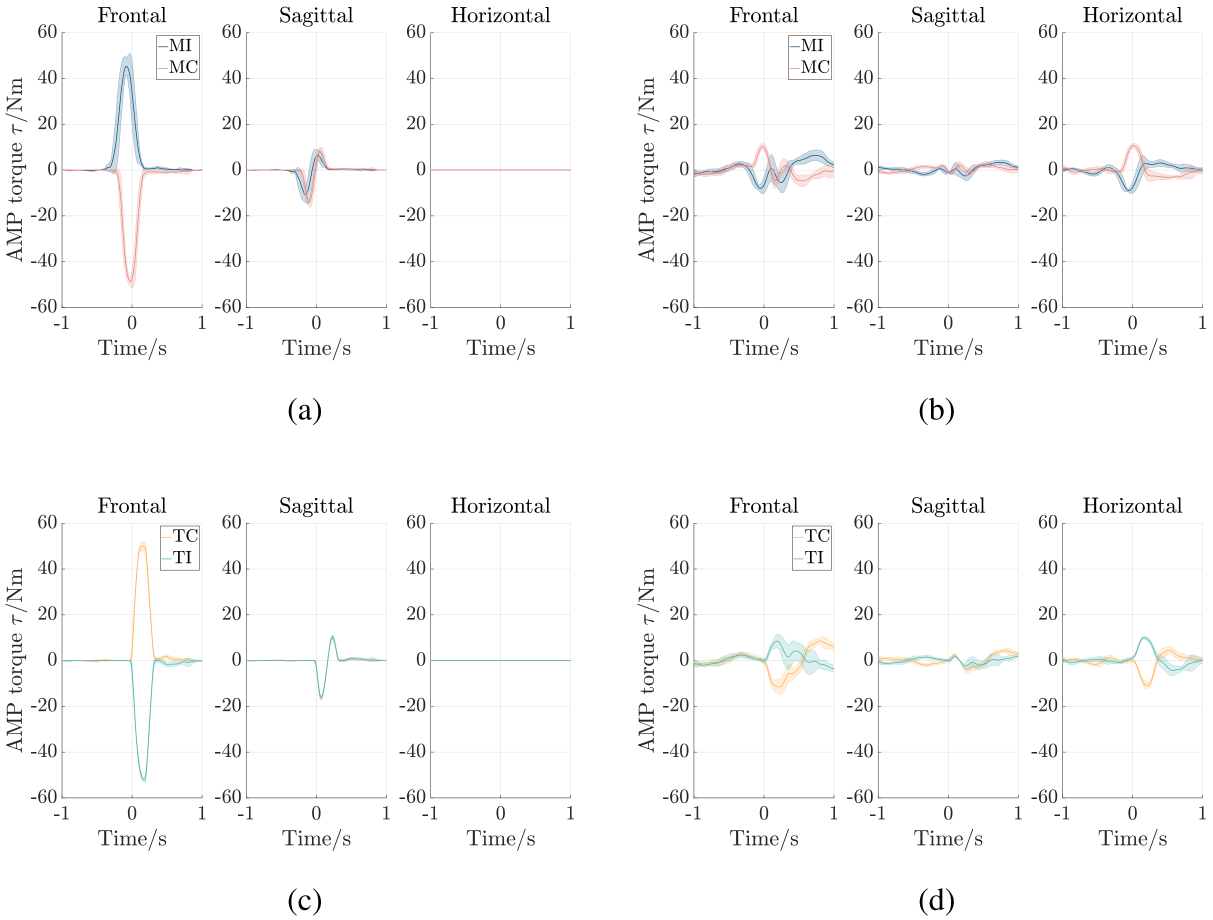

Figure S4: Grand mean±SD of AMP output torque for all four perturbation cases in frontal, sagittal, and horizontal planes across 10 participants. (a,c) pure AMP output moment generated as a result of changes in the flywheels' angular momentum; (b,d) induced gyro output as a result of rotation from the wearer's trunk. Time 0 indicates the moment when the left leg touches the second force plate. *MI*: Midstance-Ipsilateral, *MC*: Midstance-Contralateral, *TC*: Touchdown-Contralateral, *TI*: Touchdown-Ipsilateral.

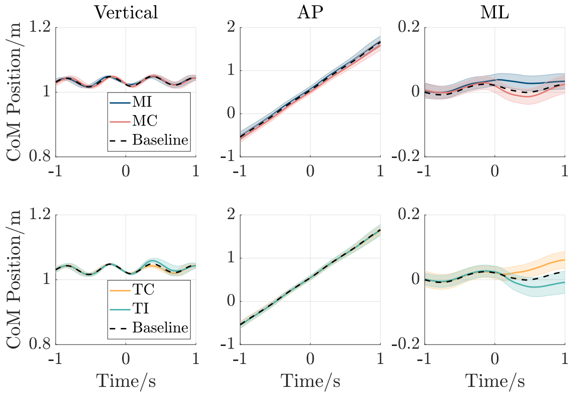

Figure S5: Grand mean±std of CoM position for all four perturbation cases in vertical, AP, and ML directions across 10 participants. Time 0 indicates the moment when the left leg touches the second force plate. The dashed black graph shows the grand average of Control Trials in the absence of any perturbation. *MI*: Midstance-Ipsilateral, *MC*: Midstance-Contralateral, *TC*: Touchdown-Contralateral, *TI*: Touchdown-Ipsilateral.

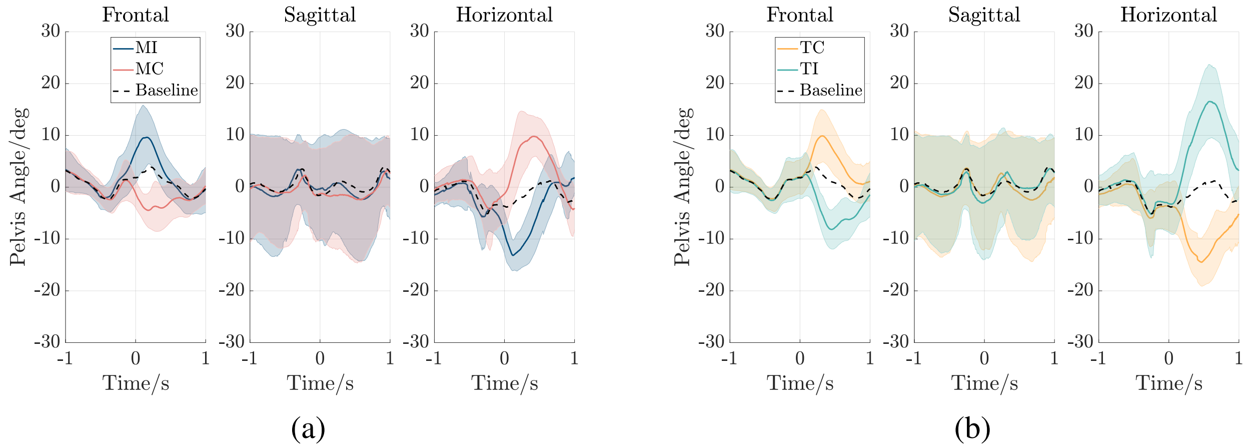

Figure S6: Grand mean±std of the pelvis for all four perturbation cases in the frontal, sagittal, and horizontal planes across 10 participants. Time 0 indicates the moment when the left leg touches the second force plate. The dashed black graph shows the grand average of Control Trials in the absence of any perturbation. *MI*: Midstance-Ipsilateral, *MC*: Midstance-Contralateral, *TC*: Touchdown-Contralateral, *TI*: Touchdown-Ipsilateral.

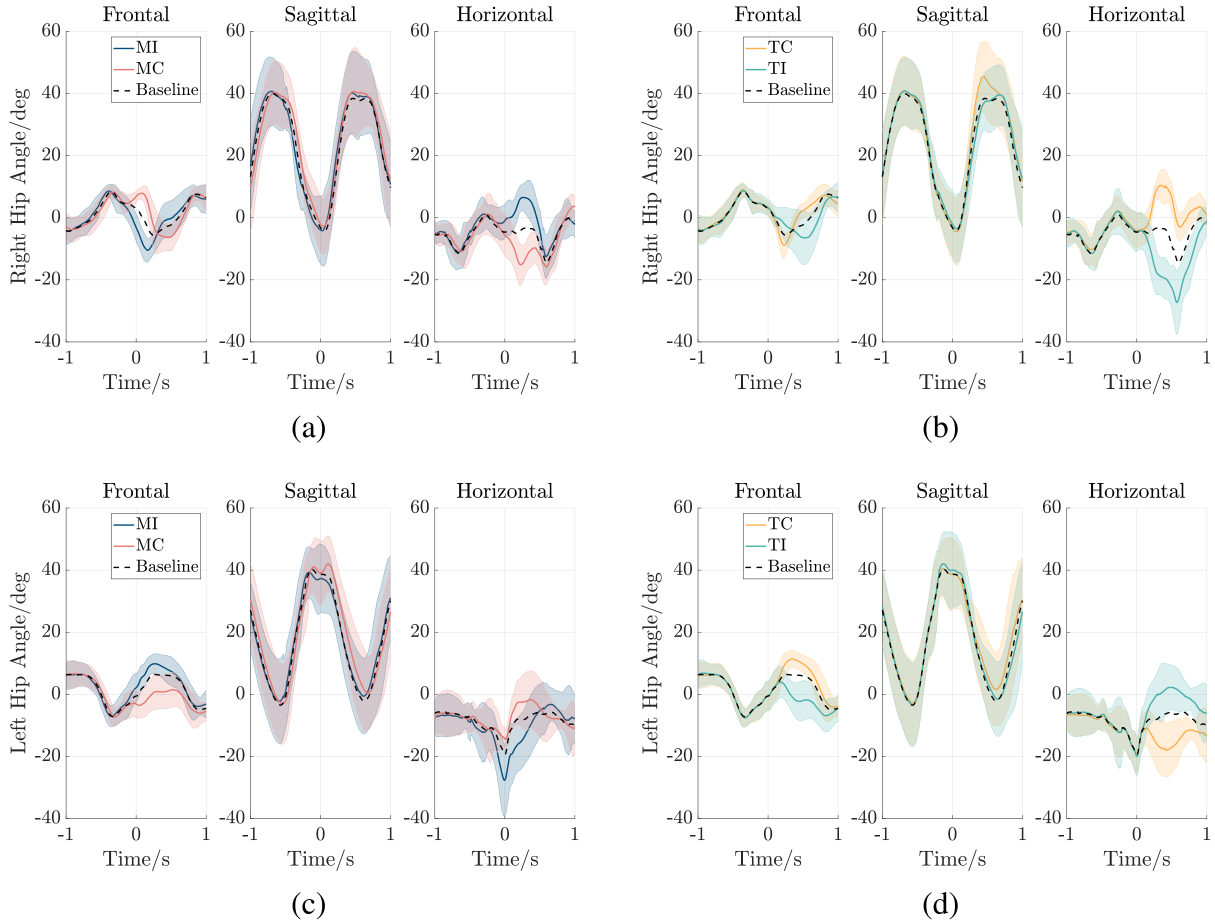

Figure S7: Grand mean±std of the (a,b) right and (c,d) left hip joint for all four perturbation cases in the frontal, sagittal, and horizontal planes across 10 participants. Time 0 indicates the moment when the left leg touches the second force plate. The dashed black graph shows the grand average of Control Trials in the absence of any perturbation. *MI*: Midstance-Ipsilateral, *MC*: Midstance-Contralateral, *TC*: Touchdown-Contralateral, *TI*: Touchdown-Ipsilateral.

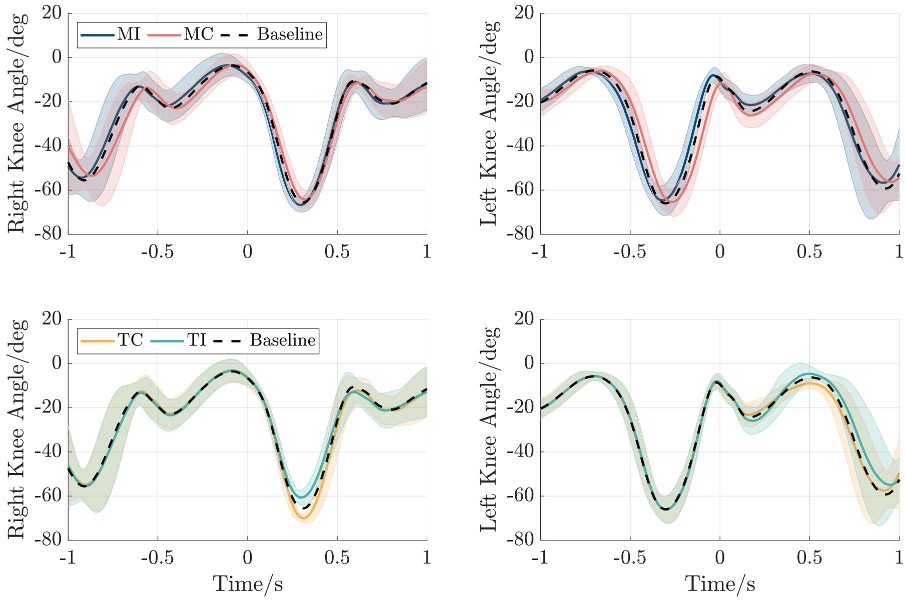

Figure S8: Grand mean±std of the right (first column) and left (second column) knee joint for all four perturbation cases in the frontal, sagittal, and horizontal planes across 10 participants. Time 0 indicates the moment when the left leg touches the second force plate. The dashed black graph shows the grand average of Control Trials in the absence of any perturbation. *MI*: Midstance-Ipsilateral, *MC*: Midstance-Contralateral, *TC*: Touchdown-Contralateral, *TI*: Touchdown-Ipsilateral.

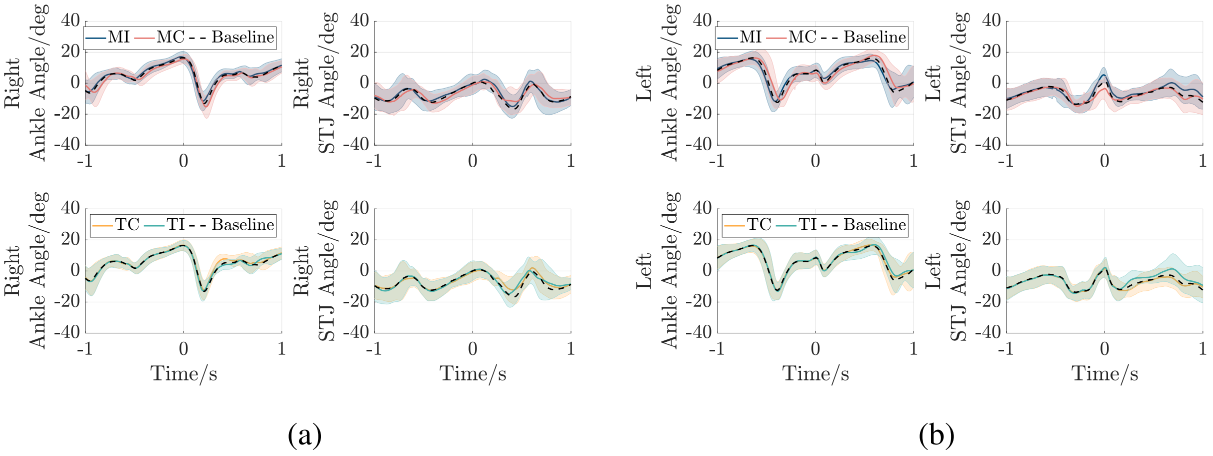

Figure S9: Grand mean±std of the (a) right and (b) left ankle joint and subtalar joint (STJ) angle for all four perturbation cases in the frontal, sagittal, and horizontal planes across 10 participants. Time 0 indicates the moment when the left leg touches the second force plate. The dashed black graph shows the grand average of Control Trials in the absence of any perturbation. *MI*: Midstance-Ipsilateral, *MC*: Midstance-Contralateral, *TC*: Touchdown-Contralateral, *TI*: Touchdown-Ipsilateral.

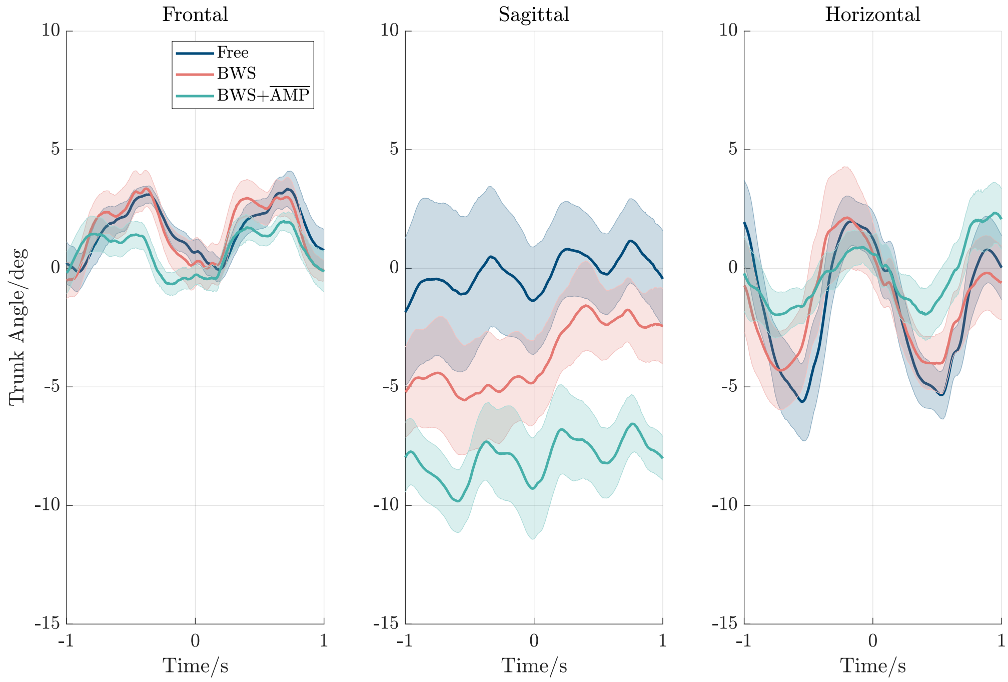

Figure S10: Comparison of trunk angle among the experimental cases labeled as ‘Free,’ ‘BWS,’ and ‘BWS+$\overline{\text{AMP}}$’ in three directions: frontal, sagittal, and horizontal planes. Time 0 indicates the moment when the left leg touches the second force plate.

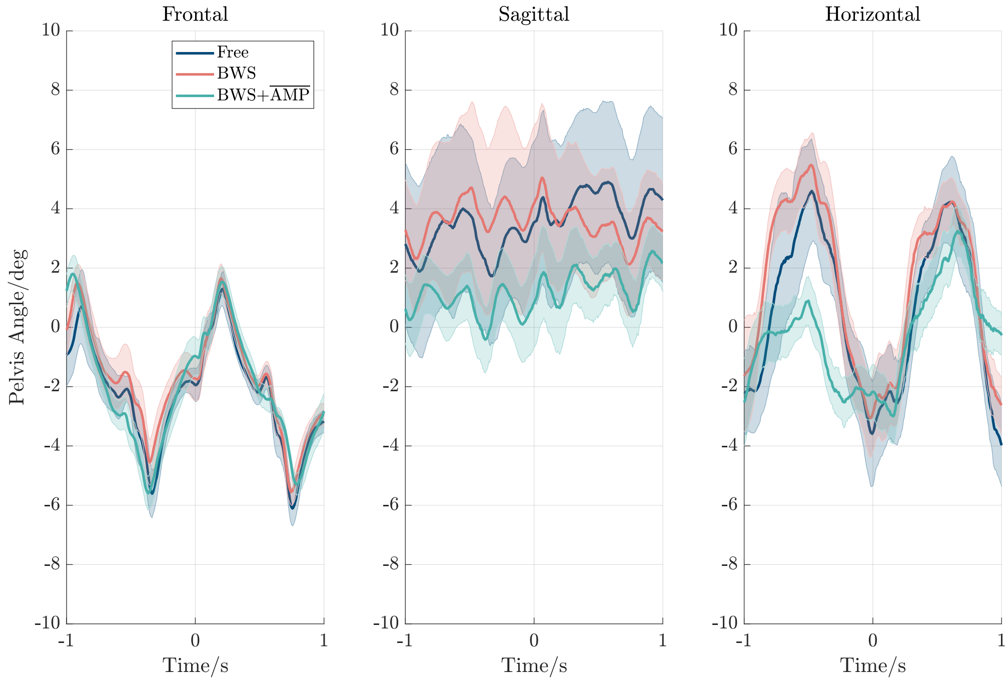

Figure S11: Comparison of pelvis angle among the experimental cases labeled as ‘Free,’ ‘BWS,’ and ‘BWS+$\overline{\text{AMP}}$’ in three directions: frontal, sagittal, and horizontal planes. Time 0 indicates the moment when the left leg touches the second force plate.

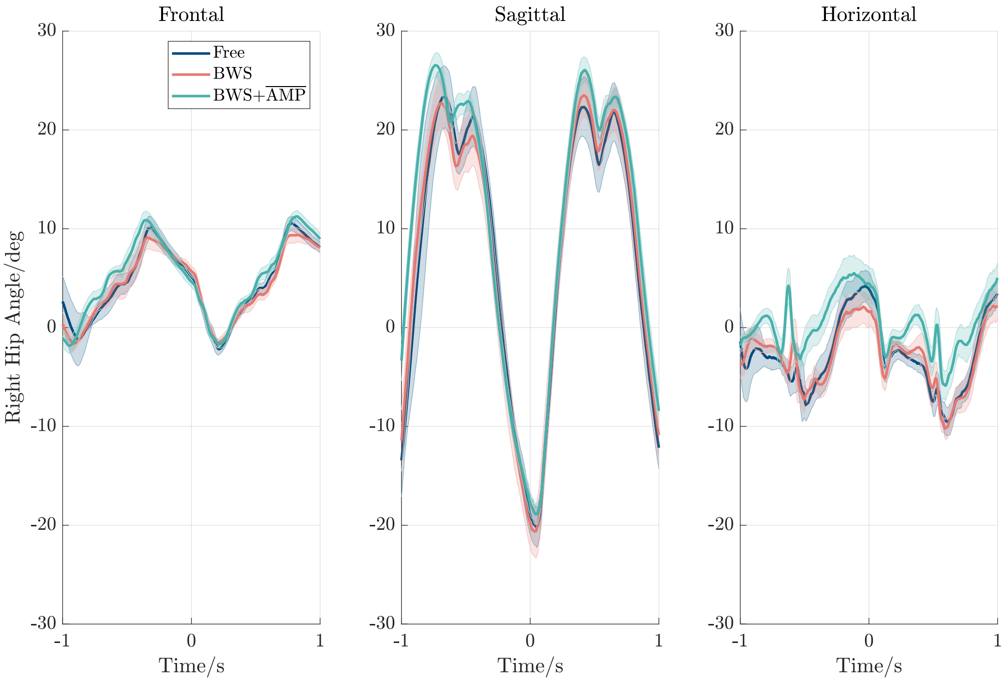

(a)

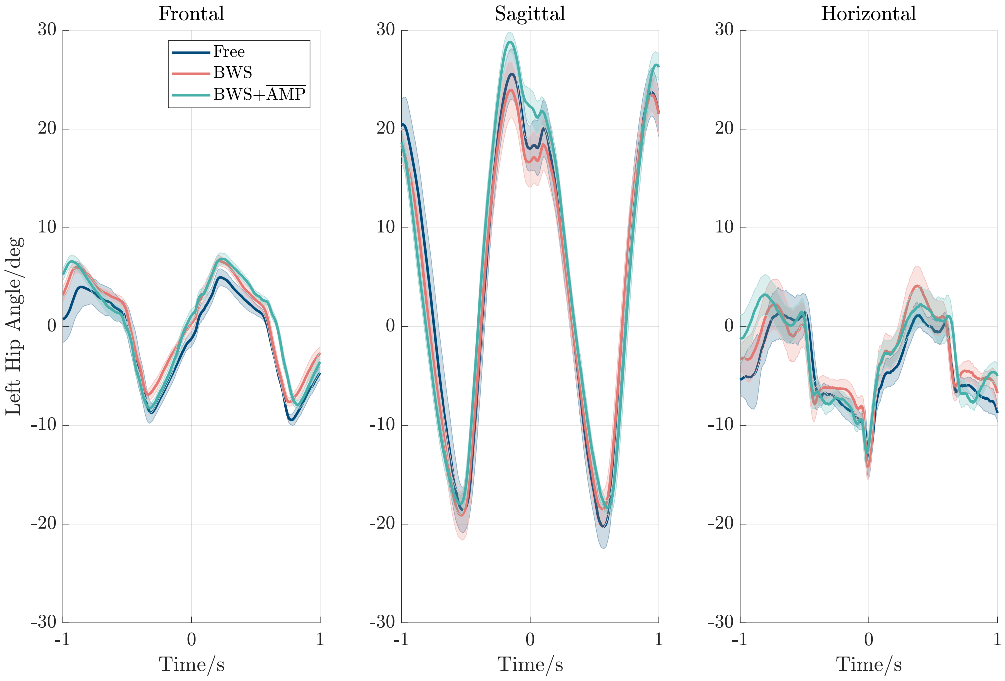

(b)

Figure S12: Comparison of (a) right and (b) left hip angle among the experimental cases labeled as ‘Free,’ ‘BWS,’ and ‘BWS+$\overline{\text{AMP}}$’ in three directions: frontal, sagittal, and horizontal planes. Time 0 indicates the moment when the left leg touches the second force plate.

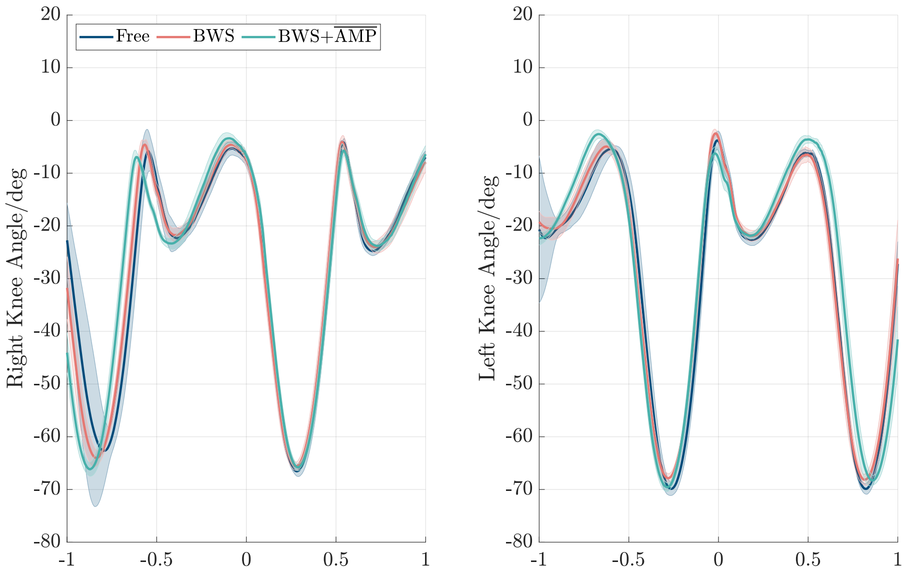

Figure S13: Comparison of knee angle among the experimental cases labeled as 'Free,' 'BWS,' and 'BWS+$\overline{\text{AMP}}$' in three directions: frontal, sagittal, and horizontal planes. Time 0 indicates the moment when the left leg touches the second force plate.

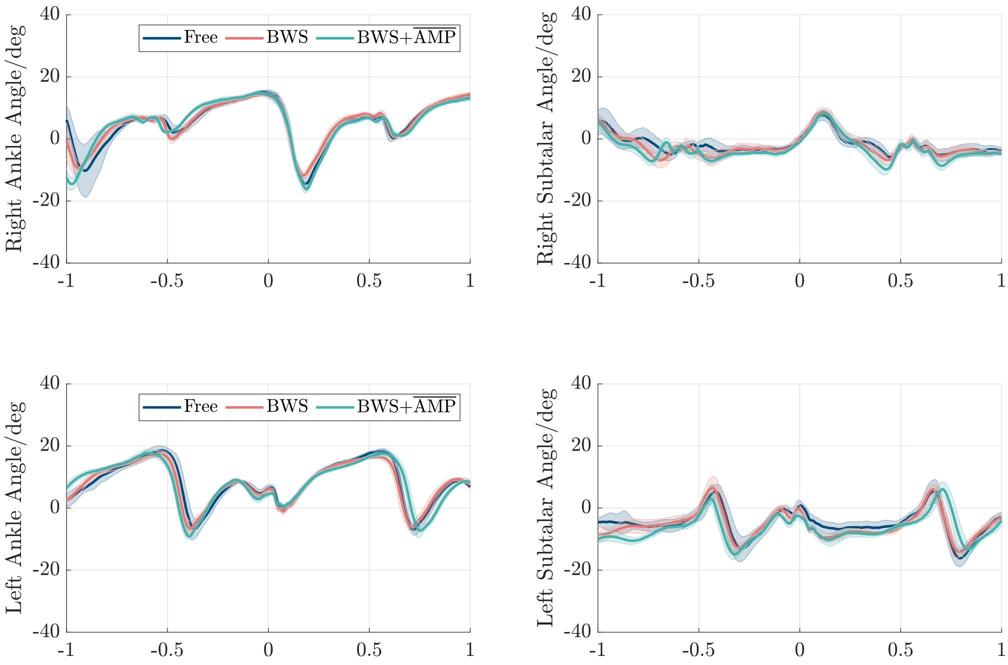

Figure S14: Comparison of (a) right and (b) left ankle angle among the experimental cases labeled as 'Free,' 'BWS,' and 'BWS+$\overline{\text{AMP}}$' in three directions: frontal, sagittal, and horizontal planes. Time 0 indicates the moment when the left leg touches the second force plate.

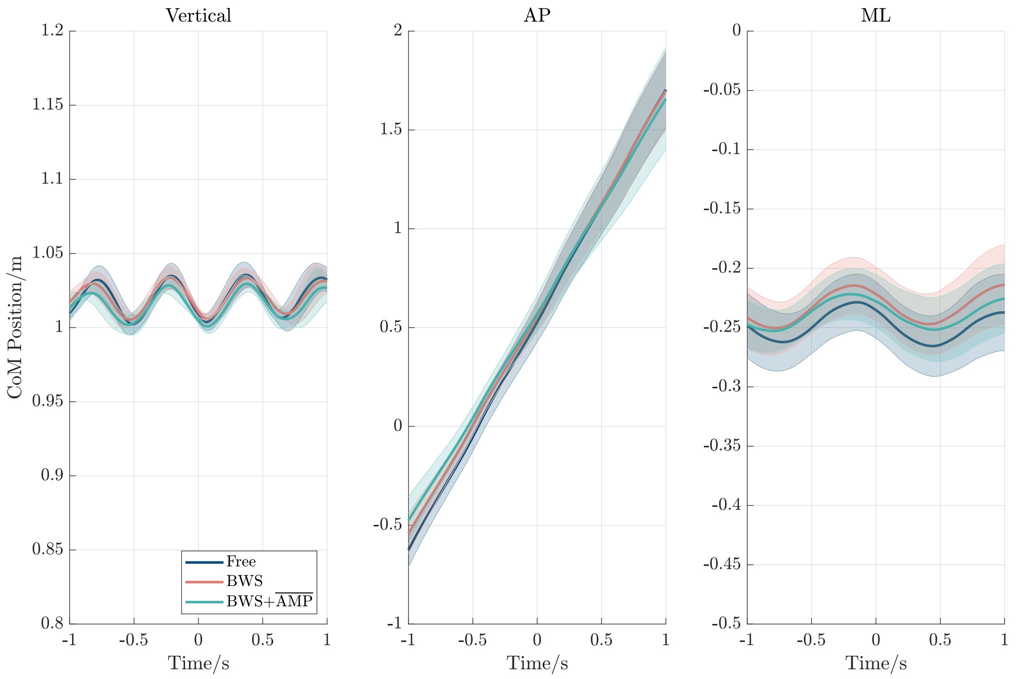

(a)

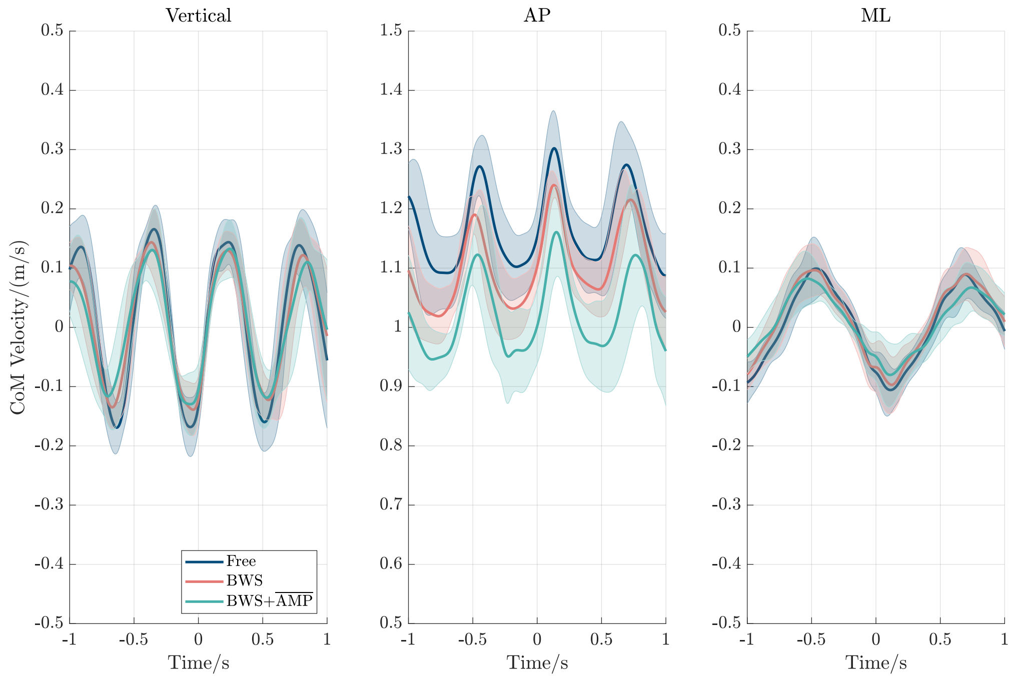

(b)

Figure S15: Comparison of (a) position and (b) velocity of CoM among the experimental cases labeled as ‘Free,’ ‘BWS,’ and ‘BWS+$\overline{\text{AMP}}$’ in vertical, AP, and ML directions across 10 participants. Time 0 indicates the moment when the left leg touches the second force plate.

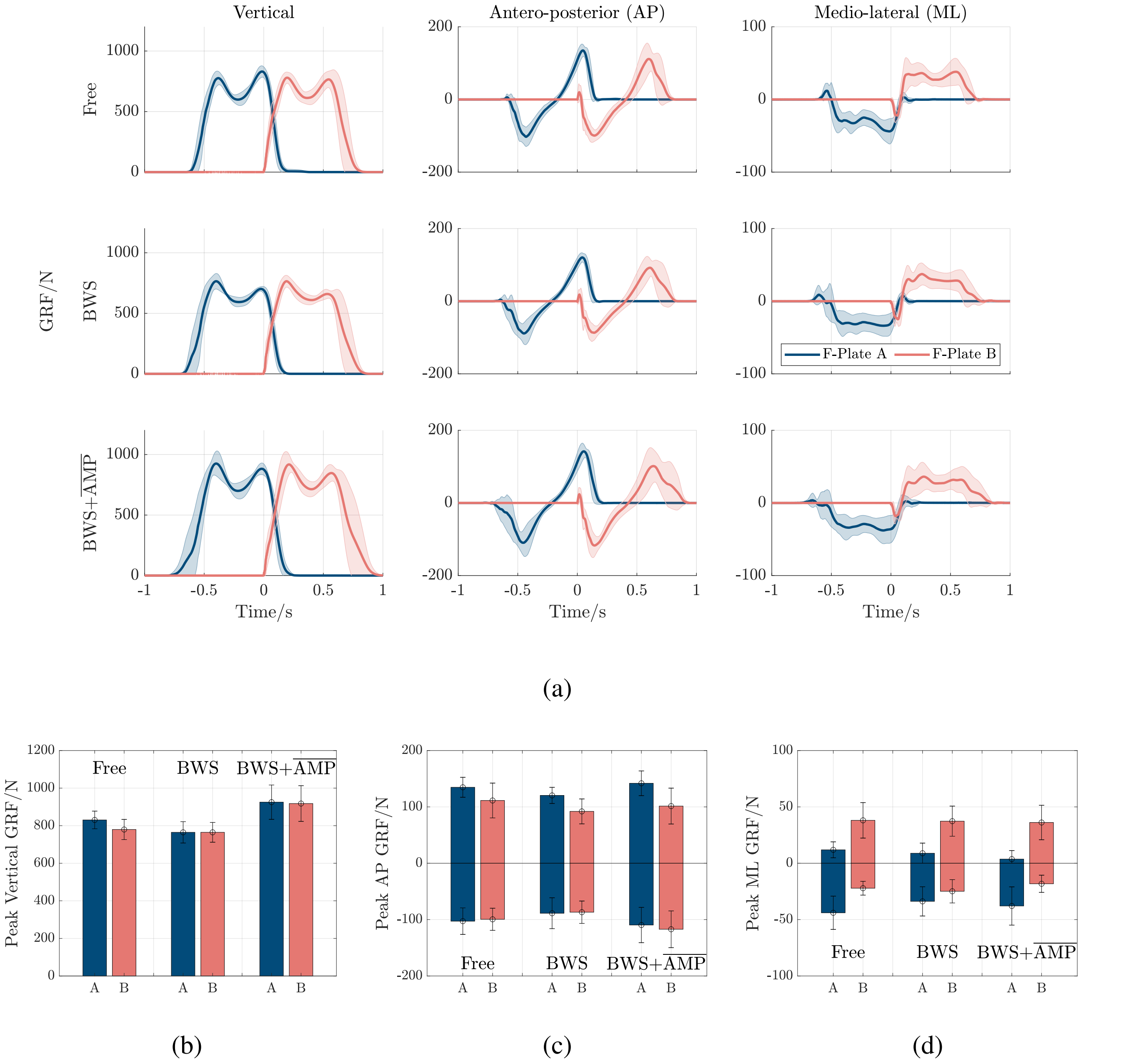

Figure S16: Comparison of ground reaction force (GRF) patterns and peaks among the experimental cases labeled as 'Free,' 'BWS,' and 'BWS+$\overline{\text{AMP}}$' in three directions: Vertical, Anteroposterior (AP), and Mediolateral (ML). (a) Grand mean±std of GRF for the three experimental cases across 10 participants. The blue and red graphs correspond to the output from force plates A and B, respectively. All participants have the same limb preference and initiate the step on the first force plate (A) with their right leg. (b,c,d) Peak±std of GRF for the three experimental cases and in each of the three directions.

Table S1. Relative mean absolute value (MAV) and p-values of eight lower-limb muscles for the perturbation stride and the subsequent stride. Statistical analysis employed linear mixed-effects models (with fixed slope and random intercept), with correction for multiple comparisons using the false discovery rate (FDR) method. Significant values ($p < 0.05$) are highlighted in bold. *MI*: Midstance-Ipsilateral, *MC*: Midstance-Contralateral, *TC*: Touchdown-Contralateral, *TI*: Touchdown-Ipsilateral.

		Perturbation Stride				Subsequent Stride			
		Left		Right		Left		Right	
		MAV %	p-value	MAV %	p-value	MAV %	p-value	MAV %	p-value
MI	GLU	16.50	**0.0281**	22.54	0.1783	5.18	0.7644	14.17	0.2489
	TFL	16.00	0.0667	11.68	0.4751	14.59	**0.0438**	14.17	0.6722
	HAM	19.25	0.0573	26.78	0.0715	1.83	0.7644	20.22	0.0728
	RF	14.16	**0.0281**	25.85	**0.0303**	12.94	**0.0438**	10.51	0.6838
	VL	3.62	0.7644	12.15	**0.0049**	0.42	0.5224	7.84	0.3640
	GAS	8.52	0.1135	19.78	**0.0303**	-7.41	0.0667	13.21	0.0619
	SOL	7.11	0.0677	15.77	**0.0303**	2.60	0.3600	7.52	0.1783
	TA	1.64	0.6813	10.34	0.0518	5.15	**0.0438**	-2.07	0.5158
MC	GLU	32.20	0.1850	10.50	0.3177	-4.93	0.3189	17.15	**0.0284**
	TFL	34.02	0.1850	32.72	**0.0284**	3.16	0.3189	18.92	0.3469
	HAM	37.49	0.1850	13.55	0.3177	-2.00	0.7049	20.51	**0.0284**
	RF	35.38	**0.0168**	18.14	0.3469	-4.04	0.5731	16.49	0.3469
	VL	39.75	**0.0000**	12.10	**0.0284**	-4.86	0.1950	-1.82	0.7915
	GAS	-0.85	0.7955	0.38	0.9055	1.09	0.7955	9.93	0.1376
	SOL	-0.07	0.3189	4.84	0.3620	-2.48	0.8032	4.09	0.3620
	TA	13.71	**0.0181**	6.28	0.3469	1.78	0.5686	-2.87	0.6206
TC	GLU	10.44	0.6591	2.89	0.4500	5.15	0.8683	19.80	**0.0006**
	TFL	46.97	**0.0160**	19.49	**0.0359**	17.20	**0.0197**	17.03	0.8698
	HAM	12.21	0.4716	3.43	0.7424	-0.08	0.6591	28.75	**0.0273**
	RF	28.68	**0.0030**	21.18	0.1129	12.33	**0.0354**	8.52	0.7543
	VL	15.67	**0.0160**	5.57	0.4500	-1.02	0.9113	1.17	0.7817
	GAS	7.82	0.0740	6.97	0.2578	-5.27	0.4716	19.30	**0.0273**
	SOL	3.42	0.6591	6.15	0.1477	2.71	0.4731	7.17	0.5000
	TA	19.01	0.0740	6.23	0.1718	5.07	0.2557	-2.80	0.5889
TI	GLU	29.51	0.4213	9.13	0.9906	5.79	0.6323	16.46	0.2242
	TFL	30.55	**0.0314**	10.50	0.9906	11.09	0.0882	12.48	0.6387
	HAM	41.84	**0.0298**	34.73	0.1976	5.97	0.1569	21.06	0.0985
	RF	60.75	**0.0004**	3.74	0.9906	-4.50	0.4351	45.73	0.3941
	VL	26.31	**0.0015**	3.99	0.6387	1.18	0.5257	11.90	0.6387
	GAS	2.62	0.5257	5.48	0.7318	11.17	0.0651	16.64	0.0985
	SOL	5.28	0.2963	0.64	0.9906	2.81	0.1672	5.98	0.6387
	TA	30.58	**0.0015**	0.05	0.9906	8.87	0.0710	-1.98	0.9837

Table S2. Means and standard deviations (SD) of onset latency (ms) of lower-limb muscles in response to mediolateral gyroscopic moment perturbations. Zero values for the SD indicate that the onset latency was solely related to one participant. To ensure consistency in the observed muscle recruitment pattern across all individuals, results derived from fewer than three participants were excluded from the analysis. *MI*: Midstance-Ipsilateral, *MC*: Midstance-Contralateral, *TC*: Touchdown-Contralateral, *TI*: Touchdown-Ipsilateral.

	Onset Latency											
	Left						Right					
		Mean	SD		Mean	SD		Mean	SD		Mean	SD
MI	GLU	303	110	VL	292	128	GLU	246	56	VL	202	21
	TFL	209	0	GAS	307	44	TFL	257	82	GAS	217	81
	HAM	232	94	SOL	271	62	HAM	212	65	SOL	204	143
	RF	146	0	TA	348	117	RF	191	20	TA	180	61
MC	GLU	202	9	VL	251	79	GLU	166	29	VL	223	54
	TFL	199	56	GAS	468	254	TFL	164	0	GAS	396	25
	HAM	203	18	SOL	185	53	HAM	252	104	SOL	385	173
	RF	261	85	TA	264	58	RF	247	87	TA	247	116
TC	GLU	295	48	VL	251	59	GLU	266	0	VL	122	37
	TFL	160	21	GAS	263	42	TFL	194	0	GAS	232	97
	HAM	267	81	SOL	349	38	HAM	194	49	SOL	197	61
	RF	191	32	TA	189	9	RF	236	0	TA	257	107
TI	GLU	257	111	VL	251	63	GLU	265	129	VL	146	35
	TFL	250	153	GAS	187	79	TFL	273	200	GAS	309	23
	HAM	247	107	SOL	171	5	HAM	266	44	SOL	292	176
	RF	162	56	TA	184	18	RF	173	3	TA	195	14

Table S3. Statistical analysis of onset latency between proximal and distal lower-limb muscles in response to upper-body gyroscopic perturbations. Statistical analysis employed linear mixed-effects models (with fixed slope and random intercept), with correction for multiple comparisons using the false discovery rate (FDR) method. Significant values ($p < 0.05$) are highlighted in bold. To ensure consistency in the observed muscle recruitment pattern across all individuals, results derived from fewer than three participants were excluded from the analysis. *MI*: Midstance-Ipsilateral, *MC*: Midstance-Contralateral, *TC*: Touchdown-Contralateral, *TI*: Touchdown-Ipsilateral.

Statistical Comparison										
	Left					Right				
	p-values					p-values				
		VL	GAS	SOL	TA		VL	GAS	SOL	TA
MI	GLU	0.7031	0.8456	0.4106	0.4052	GLU	0.8457	0.8523	-	0.8457
	TFL	-	-	-	-	TFL	0.6947	0.8457	-	0.6947
	HAM	0.2123	0.0732	0.4052	**0.0369**	HAM	0.8457	0.8457	-	0.8457
	RF	-	-	-	-	RF	0.8457	0.6947	-	0.8457
MC	GLU	0.4600	-	0.9934	0.4600	GLU	0.4591	**0.0057**	**0.0057**	0.4591
	TFL	-	-	-	-	TFL	-	-	-	-
	HAM	0.4600	-	0.9934	0.4600	HAM	0.9947	**0.0391**	**0.0391**	0.9947
	RF	0.9934	-	0.4600	0.9934	RF	0.9947	**0.0391**	**0.0391**	0.9947
TC	GLU	0.2749	0.3835	0.2219	**0.0323**	GLU	-	-	-	-
	TFL	-	-	-	-	TFL	-	-	-	-
	HAM	0.0635	**0.0323**	**0.0005**	0.5550	HAM	-	0.8405	0.7514	0.8848
	RF	0.1808	0.0788	**0.0010**	0.9746	RF	-	-	-	-
TI	GLU	0.9385	0.5674	0.5674	0.5674	GLU	0.2080		0.6621	0.3715
	TFL	-	-	-	-	TFL	-	-	-	-
	HAM	0.9385	0.5674	0.5674	0.5674	HAM	0.2080	-	0.6621	0.3715
	RF	0.5674	0.9385	0.9385	0.9385	RF	-	-	-	-

REFERENCES

Schumacher, C., Berry, A., Lemus, D., Rode, C., Seyfarth, A., and Vallery, H. (2019). Biarticular muscles are most responsive to upper-body pitch perturbations in human standing. *Sci Rep* 9, 1–14. doi:10.1038/s41598-019-50995-3
